# Context matters during pick-and-place in VR: Impact on search and transport phases

**DOI:** 10.3389/fpsyg.2022.881269

**Published:** 2022-09-08

**Authors:** Olga Lukashova-Sanz, Rajat Agarwala, Siegfried Wahl

**Affiliations:** ^1^Zeiss Vision Science Lab, Institute for Ophthalmic Research, University of Tübingen, Tübingen, Germany; ^2^Carl Zeiss Vision International Gesellschaft mit beschränkter Haftung (GmbH), Aalen, Germany

**Keywords:** pick-and-place task, scene context, object manipulation, virtual reality, realistic scene

## Abstract

When considering external assistive systems for people with motor impairments, gaze has been shown to be a powerful tool as it is anticipatory to motor actions and is promising for understanding intentions of an individual even before the action. Up until now, the vast majority of studies investigating the coordinated eye and hand movement in a grasping task focused on single objects manipulation without placing them in a meaningful scene. Very little is known about the impact of the scene context on how we manipulate objects in an interactive task. In the present study, it was investigated how the scene context affects human object manipulation in a pick-and-place task in a realistic scenario implemented in VR. During the experiment, participants were instructed to find the target object in a room, pick it up, and transport it to a predefined final location. Thereafter, the impact of the scene context on different stages of the task was examined using head and hand movement, as well as eye tracking. As the main result, the scene context had a significant effect on the search and transport phases, but not on the reach phase of the task. The present work provides insights into the development of potential supporting intention predicting systems, revealing the dynamics of the pick-and-place task behavior once it is realized in a realistic context-rich scenario.

## 1. Introduction

Over the last several decades, the development of external assistive systems, such as prosthetic arms and exoskeletons, received much attention due to their strong potential to complement and improve the lives of people with sensory-motor impairments (Lazarou et al., 2018). One of the grand challenges for these assistive means is non-intuitive complicated mutual communication between the device and the patient, and thus, their inability to adapt to the individual patient's needs (Lazarou et al., 2018; Sensinger and Dosen, 2020). The evolution of the eye-tracking technology opened a possibility to ensure more intuitive communication between the assistive system and the user (Shafti et al., 2019; de Brouwer et al., 2021; Subramanian et al., 2021). Developing an intuitive algorithm that uses eye movements to support the user in object manipulation requires the knowledge of how our eyes and hand move when grasping an object. Several studies investigated the dynamics of object manipulation. They described the coordinated eye and hand movements in a grasping task for intact people as well as people with motor disabilities (Ballard et al., 1992; Johansson et al., 2001; Lavoie et al., 2018; Gregori et al., 2019; de Brouwer et al., 2021). Even so, the vast majority of the research on eye-hand interaction in a grasping task mainly focused on single objects manipulation without placing them in a meaningful scene (e.g., Lavoie et al., 2018; Gregori et al., 2019). In real life, however, we perceive the world and interact with the surrounding objects in a context-rich environment rather than isolated objects (Võ and Wolfe, 2013; Võ, 2021). While the existing research provides valuable information about eye and hand movement when performing a grasp of an object and transporting it from one location to another, it is still unclear whether the eye and hand movement will remain in a more realistic scene context the same. It is, therefore, essential to investigate human object manipulation once the object is placed into a context-rich natural setting.

The importance of contextual information in various visual tasks has been shown in multiple studies (e.g., Chun and Jiang, 1998; Marek and Pollmann, 2020). Furthermore, the visual search research provides strong evidence that the scene context affects where and how often we gaze within a given scene (Brockmole et al., 2006; Torralba et al., 2006; Võ and Wolfe, 2013; Wolfe, 2020; Võ, 2021). As such, if we are to find a laptop in the room, we are likely to gaze mostly at the surfaces, particularly the working table (Pereira and Castelhano, 2019; Võ, 2021). Interestingly, this holds also when the search target is not present in the scene, underlining the predictive nature of human search strategy (Biederman et al., 1982; Bar, 2004, 2009; Võ et al., 2019). Scene context also has been shown to facilitate action recognition (Wurm and Schubotz, 2017). Moreover, it has been shown that when searching for an object, people tend to rely on relevant anchors in the scene, that is, larger characteristic objects which typically are associated with the target object location (Draschkow and Võ, 2017; Võ, 2021). Until now, most of the studies in the field have been implemented in a non-interactive manner, where the observer had to perform a visual search task without manipulating the target object. In real-world scenarios, however, we often do not just observe the environment but perceive the world in terms of affordances, i.e., opportunities to interact with the surrounding environment (Gibson, 2014). This interaction is tightly bound to grasping and manipulating objects around us. In a grasping task, it is yet unknown whether different phases of object manipulation, such as reaching the object or transporting it from one location to another, are affected by the scene context.

One recent study looked into the effect of the scene context consistency on interaction with objects, where participants had to construct environments from a set of virtual objects either in agreement with their semantic expectations or against them (Draschkow and Võ, 2017). Among other results, the authors showed an increased grasping time of the object when it did not match the scene context. A possible explanation for this effect is increased decision time on where to put the object when it doesn't fit the scene context. There are countless possible locations in contrast to limited locations when the object is congruent with the scene context. Furthermore, previous studies demonstrated that reaching an object before grasping it can be affected by various factors. As an example, motor inhibition when approaching dangerous objects has been shown due to the emergence of aversive affordances (Mustile et al., 2021). These studies provide evidence that the way we look at and grasp objects around us might differ depending on the meaning of that object and the semantic context they are placed.

In daily life, we often are confronted with a combination of a visual search task and subsequent grasping of an object, such as when we are looking for the keys before leaving the house. How is the behavior in such a scenario affected by the scene context? Would there be a difference when the object fits or doesn't fit the scene context or when there is no context at all? In the present study, we addressed object manipulation in a pick-and-place task when performing it in a context-rich environment and when no meaningful context is present. In particular, the present study is intended to investigate whether the scene context primarily impacts only the searching, as is suggested in the visual search literature where the object is searched longer if it is incongruent with the scene context compared to when it fits the scene. Alternatively, when the object does not match the scene context, does it take people longer to reach or transport it in addition to a more prolonged search? Moreover, consider comparing scenarios where the target object is placed in a semantically congruent context against the case when the object is isolated, i.e., in a context-poor environment. Would the presence of additional visual stimuli and possible obstacles in the context-rich environment serve as a distractor and lead to a more prolonged search and further interactions with the object even when the object matches the context, or would the scene context facilitate the search and object manipulation? To answer these questions and advance in understanding how humans manipulate objects in realistic scenes, it is vital to systematically address the effect of the scene context on different phases of our interaction with objects.

When studying interactive object manipulation, it is important to develop the paradigm realistically. Specifically, unconstrained head and hand movement and free eye movement are essential for natural behavior. The rapid development of modern technologies such as Virtual Reality (VR) and VR eye-tracking enabled researchers to study human interaction with the surrounding environment in more realistic 3D settings (Boettcher et al., 2018; Olk et al., 2018). Furthermore, VR provides a possibility to simulate various real-world scenarios in a yet controlled laboratory environment at an efficient cost. To explore the interactive domain of object manipulation, it is thus, convenient to develop the experimental paradigm in VR. When studying grasping using VR, it can be, however, challenging to reproduce natural grasping behavior in a virtual scene due to the mismatch of the visual response and lack of haptic feedback (Levin et al., 2015; Furmanek et al., 2019). In VR experiments, typically, the interaction with the virtual environment is realized *via* controllers instead of a real hand (e.g., Draschkow and Võ, 2017), where depending on the virtual hand representation, differences in hand movement between the real and virtual settings might emerge (Viau et al., 2004; Cai et al., 2021). Nonetheless, recent studies demonstrated that a virtual representation of a hand-looking object when interacting in VR could be a reasonable approach to the imitation of a realistic grasping as it enables the strongest sense of ownership (Lougiakis et al., 2020; Cai et al., 2021; Lavoie and Chapman, 2021). The realism can be enhanced when showing grasping an object by presenting a grasping hand pose holding the object (Tian et al., 2019; Lavoie and Chapman, 2021). Combining these findings with the advantages of VR for the experimental design mentioned above, in the present study, we chose VR as the tool to study object manipulation, where a virtual glove represented the hand, and respective grasping poses were generated.

The current study investigated the effect of the scene context on human object manipulation in an interactive task in a realistic scenario. Using the head and hand movement, as well as eye-tracking, the impact of the scene context on different stages of a pick-and-place task was examined. Specifically, pick-and-place task performance was evaluated while placing the objects of interest into typical everyday visual scenes implemented in VR.

## 2. Materials and methods

### 2.1. Participants

Thirteen naïve participants (5 female and 8 male), with normal or corrected to normal vision were tested. Participants were aged between 19 and 31 years old. No formal power analysis for the sample size calculation was performed. All procedures conformed to Standard 8 of the American Psychological Association's “Ethical Principles of Psychologists and Code of Conduct (2010)”. The study was approved by the ethics committee of the Faculty of Medicine at the University of Tübingen with a corresponding ethical approval identification code 986/2020BO2. Signed informed consent was obtained from each participant before the measurements. All data were stored and analyzed in full compliance with the principles of the Data Protection Act GDPR 2016/679 of the European Union.

### 2.2. Experimental setup

#### 2.2.1. Hardware specifications

**Figure 1:**
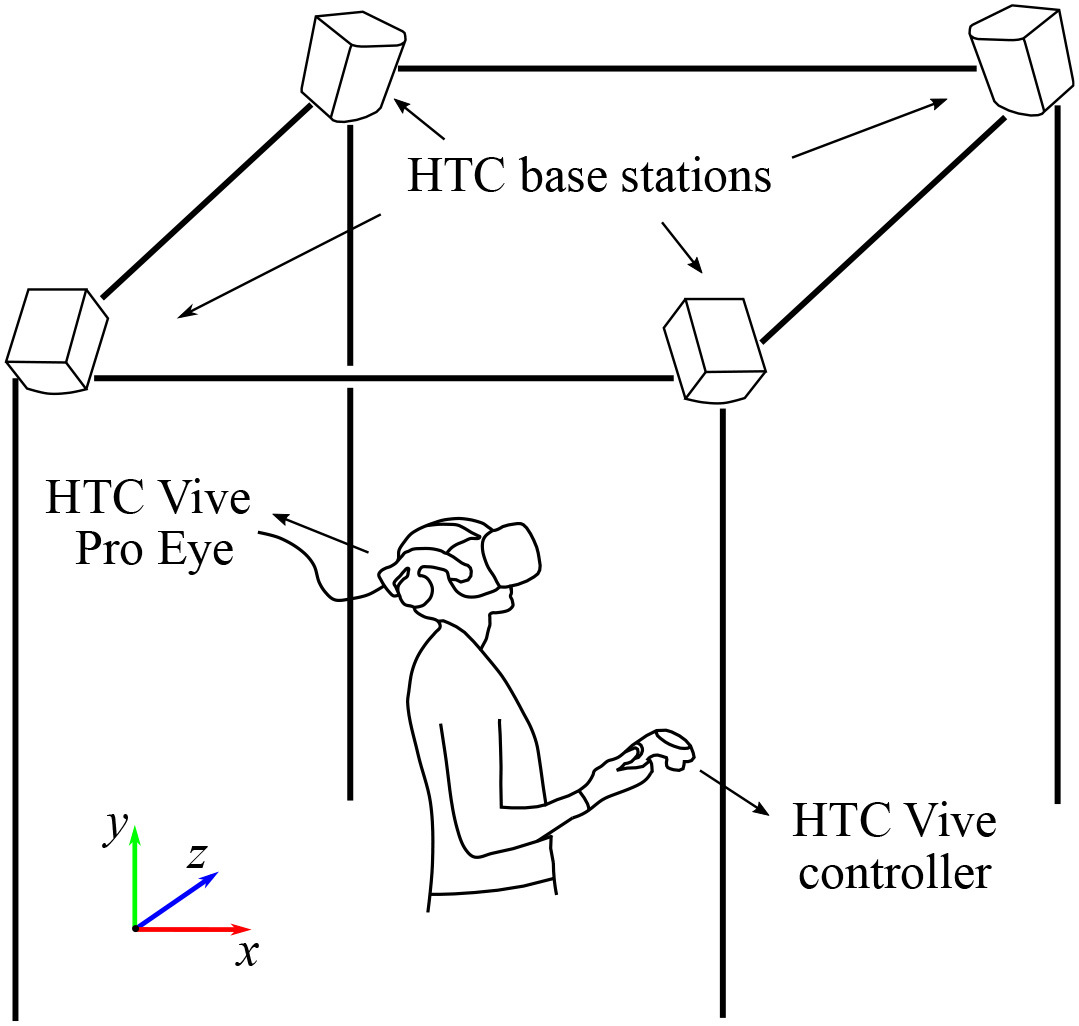
Schematic representation of the experimental setup. The visual scenes were displayed using HTC Vive Pro Eye virtual reality headset. The position and rotation of the headset and the controller were tracked *via* four HTC base stations 2.0 paced in the four corners of the working space. The interaction with the environment was realized using the HTC controller held in the right hand of the participant. The colored axes represent Unity left-hand coordinate system. For details, see the main text.

The visual content was displayed to the participants using HTC Vive Pro Eye (HTC Corporation, Taoyuan, Taiwan) virtual reality headset running on a Windows 10 PC with NVIDIA GeForce GTX 1070 graphics card (NVIDIA Corporation, Santa Clara, California, USA). The field of view of the headset and the refresh rate reported by the manufacturer are 110° and 90 Hz, respectively. The participant interacted with the environment *via* the HTC Vive controller held in the right hand of the person. The position and rotation of the headset and the controller were tracked *via* four HTC base stations 2.0. The complete size of the tracked area was approximately three by three meters. The eye-tracking data were collected using the built-in eye tracker at a frequency of 120 Hz. During the experiment, the participant was in a standing position and could freely move within the working space. The experimental setup is schematically shown in Figure 1.

#### 2.2.2. Software specifications

The experimental paradigm was generated using the Unity Game engine (Unity Technologies, 2019), Unity version 2019.4.0.f1. The eye movement data were collected using Unity package SRanipal version 1.3.3.0. Recording of the eye movement data at a maximum sampling rate 120 Hz was realized by using a separate thread parallel to the main script execution. The data analysis was performed using Python 3.6 packages NumPy (Van Der Walt et al., 2011) version 1.19.1, SciPy (Virtanen et al., 2020) version 1.5.2 and Pandas (McKinney, 2010) version 1.1.3. The statistical analysis was conducted using R version 3.6.1, in particular, package nlme (Pinheiro et al., 2022). The data visualization was performed using Python packages Matplotlib (Hunter, 2007) version 3.3.1 and Seaborn (Waskom et al., 2017) version 0.11.0.

### 2.3. Virtual environment and stimuli

#### 2.3.1. Realistic man-made VR scenes

The virtual environment was composed of realistic man-made indoor scenes. Specifically, three different habitual scene contexts were selected: kitchen, bathroom, and office. These contexts are commonly met in daily life and were previously used in the scene guidance literature (e.g., Wurm and Schubotz, 2017; Boettcher et al., 2018; Beitner et al., 2021). Two different variations of each scene context were designed, resulting in six distinct context-rich scenes. The two variations of each context were introduced to maintain the variety of the environments and unique configuration of each trial. One requirement for the scenes was an equal set of anchors (see Section 2.3.3). That is, there was a computer in each office, a microwave in each kitchen, and a sink in each bathroom. Otherwise, the room design was arbitrary. The complete set of implemented scenes can be found in Supplementary material.

**Figure 2:**
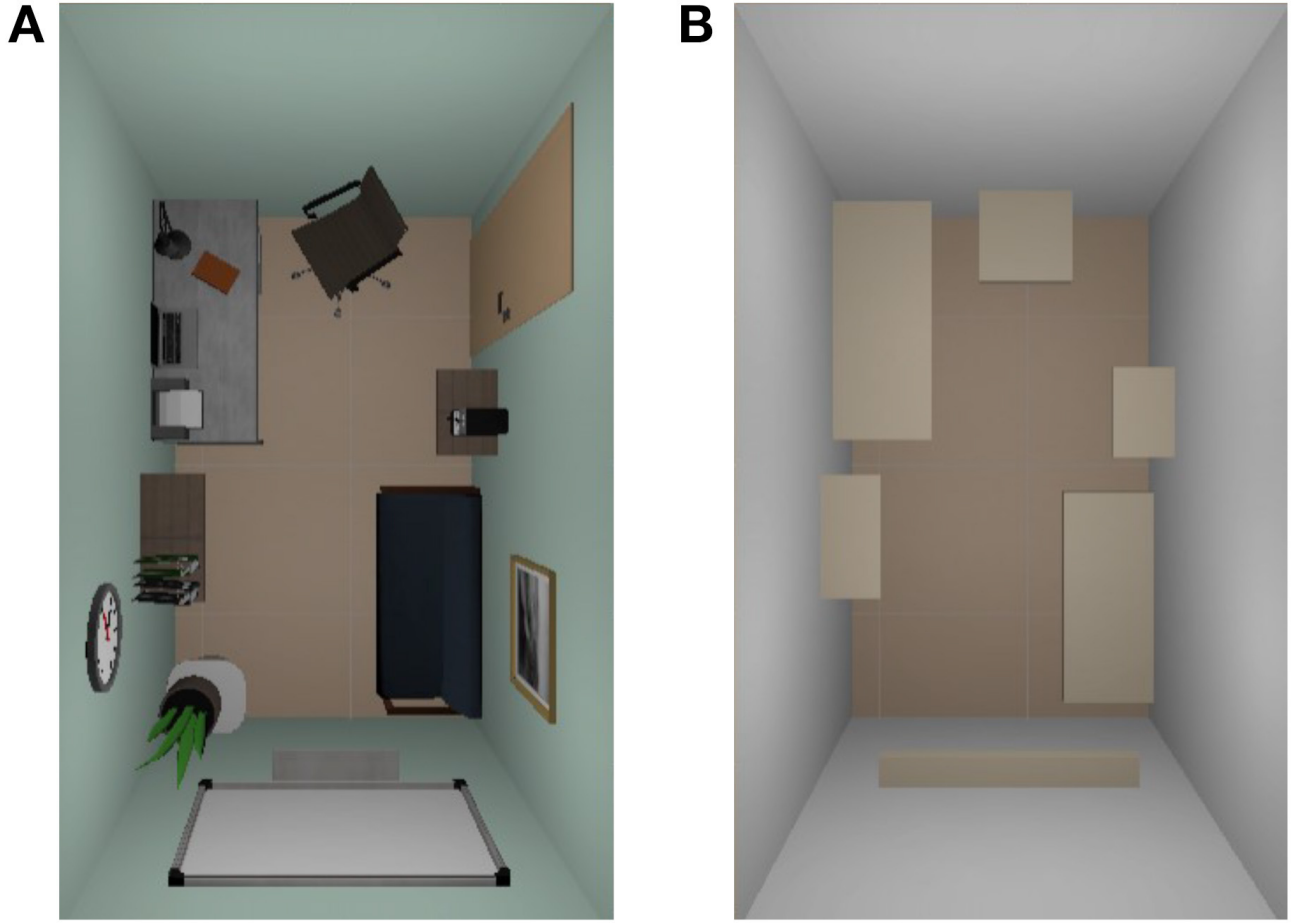
The top view of one of the designed VR scenes; **(A)** context-rich scene, here: office; **(B)** empty scene matching the spatial configuration of the respective context-rich scene. The size of the rooms was set to two by three meters. Note, the scenes are shown without the end location which was represented by a column 92 cm of height, and was always in the center of each room. The complete set of six implemented scenes and their respective empty equivalents can be found in Supplementary material.

Furthermore, six empty virtual rooms were created with no scene context present: the rooms only contained a set of shelves replicating the spatial configurations of each of the context-rich environments, respectively. Doing so, the “empty” experimental condition was implemented (for details, see Section 2.4.3). The size of the scenes was set to two by three meters. An example of one scene and its empty match is shown in Figure 2. All context-rich scenes were created using a set of open-source 3D assets. The complete set of implemented “empty” scenes can also be found in Supplementary material.

#### 2.3.2. Target objects and distractors

To maximally separate the scene context effect from such biases as the size or the shape of the object, and for comparability, we chose to use cubes with images projected on their faces instead of actual virtual objects. This way, it was intended that the participants focus more on the semantic meaning of the objects. Moreover, there is a practical advantage to using a cubic shape for a more accurate gaze evaluation. In particular, in Unity, the gaze point is detected on the object once the gaze ray hits the collider around that object (see more details in Section 2.5.3). Therefore, the collider should ideally have the same shape as the object to avoid a mismatch between detected and actual gaze points. Creating complex mesh colliders for different VR objects is a tedious task and slows down the display of the visual content in the VR headset. On the other hand, the cubic shape is one of the basic collider shapes in Unity and can be efficiently used.

**Figure 3:**
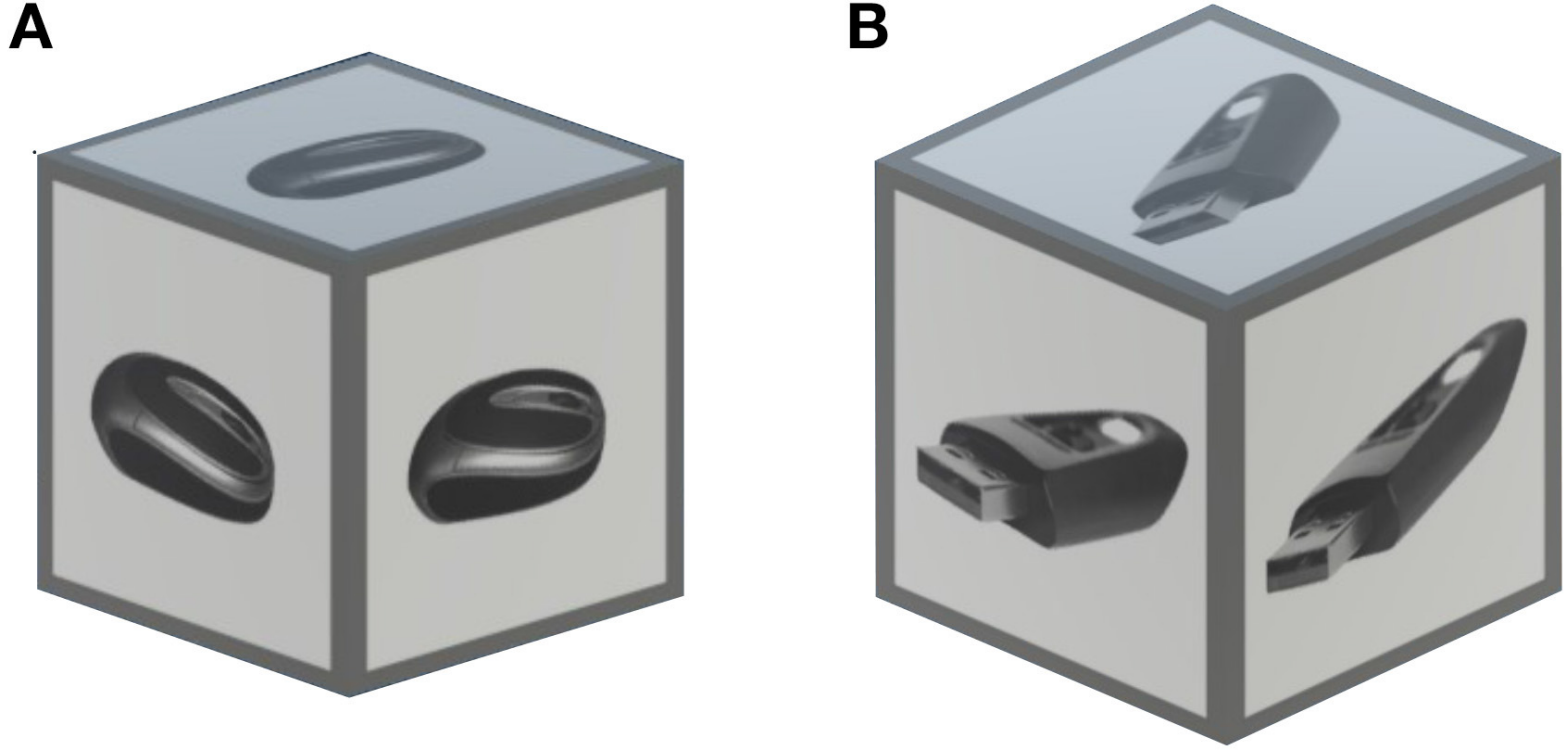
An example of **(A)** target object and **(B)** distractor used in the experiment. The objects were represented by grayscale images projected on the cube facets. The size of the cubes was set to 0.084 Unity-meters. Here, both the target object and the distractor, belong to the same scene context: office. For details, see Section 2.3.2.

A set of target objects was generated, where each object was represented by a cube with an image projection on its faces. The size of the cubes was set to 0.084 Unity-meters. For each scene context, a set of seven images was used, resulting in a total of 21 objects. All images were selected and adjusted from different open-source pictures on the Internet. The grayscale of the images was selected to avoid a pop-out effect due to the color-based saliency of some images compared to others. In Figure 3A, an example of the target object is shown. For the objects, we chose the cubic shape instead of arbitrary shapes of actual 3D objects to universalize the target objects across trials and make them comparable. Specifically, the homogeneous shape ensures no size or shape bias when estimating gaze locations on the target object. Furthermore, for the gaze evaluation, the cubic form allows a more accurate estimation of the gaze point on the object in Unity due to the simplicity of the box collider around the object.

Similarly, a set of distractors was generated for each scene context. The distractors were designed in the same manner as the target objects: grayscale images projected on the facets of cubes. The target-object-alike distractors were introduced to ensure the relative complexity of the task and prevent the participant from searching for the only cube existing in the scene. For each scene context, a set of 10 open-source images was used, resulting in a total of 10 distractors per scene. An example of a distractor is illustrated in Figure 3B.

#### 2.3.3. Anchors

For each of the context-rich scenes, a set of anchors was selected. Although there have been recent attempts to formally define anchors (Boettcher et al., 2018), up until now, no validated database was developed. Therefore, in the present study, the anchors were selected arbitrarily following an intuitive description: an anchor is considered to be a static larger object which is typically not easily moved with one hand (Võ, 2021). The complete set of the anchors and their respective target objects can be found in Supplementary material.

The predefined position for each target object was set to a location in the proximity of its anchor, where depending on the anchor, the object could appear either next to or on top of it. As such, a toothbrush would appear next to the sink in the bathroom, whereas a pan would be placed on top of the kitchen stove in the kitchen.

### 2.4. Experimental procedure

#### 2.4.1. General procedure

**Figure 4:**
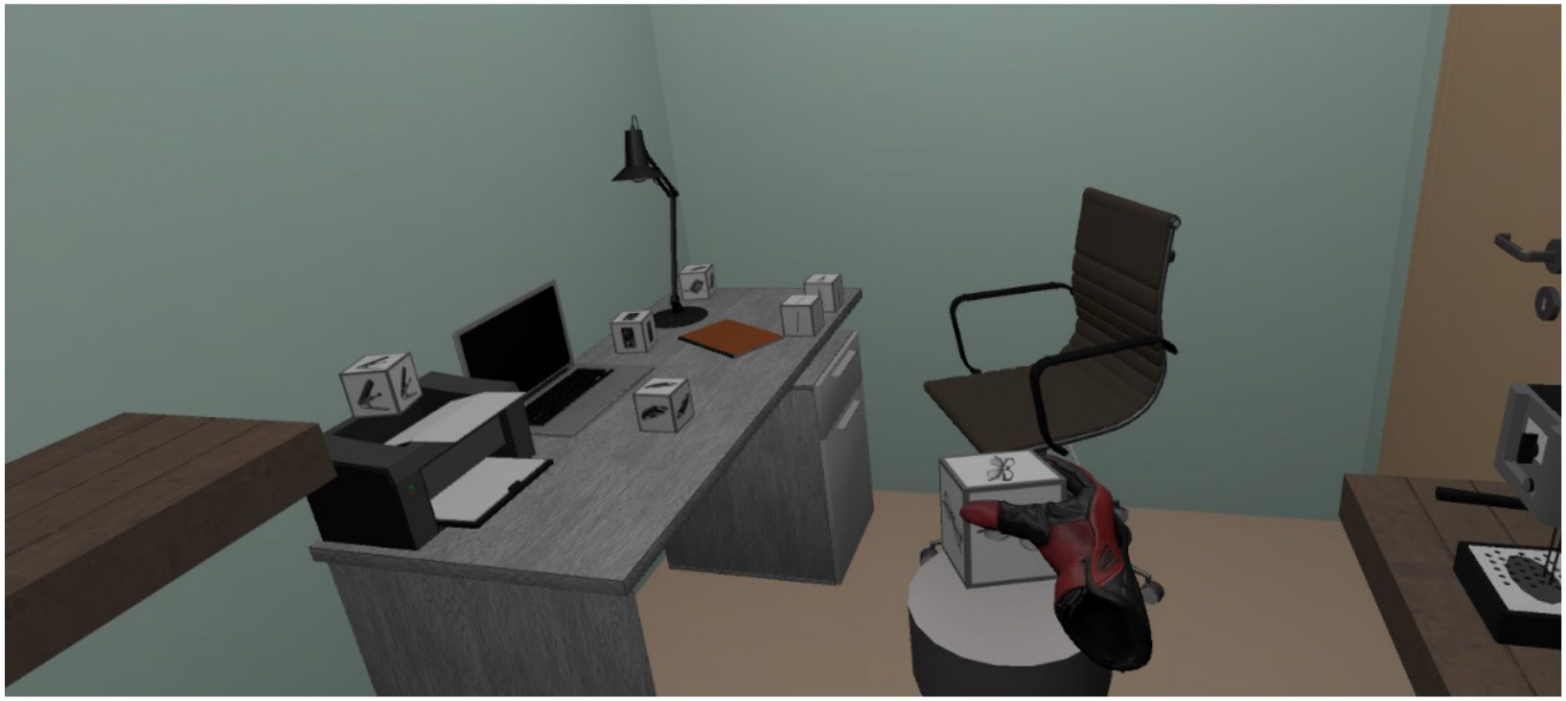
An example of a VR scene from the participant's perspective. The cubic grayscale objects are the distractors and the target object. In the lower central part of the image, a part of the end location is shown, which was represented by a column in the center of the room. The controller was represented by a virtual SteamVR glove. Note, that a grasping pose for each object was designed and generated and shown to the participant upon grasping a virtual object. For more details, see the main text.

In each trial, the participant's task was to find a specific object, pick it up using the controller, and transport the object to the final predefined location. The VR controller was represented by a SteamVR virtual glove. Note that a grasping pose for each object was designed and generated in advance and shown to the participant upon grasping a virtual object. In other words, the virtual hand did not disappear when the participant pressed the trigger to grasp the object but instead was still visible in a grasping pose (see Figure 4). The end location was represented by a column 92 cm of height and was always in the center of every room. The time of each trial was not limited, and participants were asked to perform the task at a normal pace. The participant was shown a gray background between each trial where the target object for the upcoming trial was displayed. The participant was requested to start each trial from a specific position in the room indicated by a round target which switched its color from blue to green once the participant was inside the target. To start the trial, the participant pressed a button on the controller. Thereafter, a small pause of 1 s was introduced where the participant was asked to gaze at a fixation target on the gray background before the virtual room appeared. Doing so ensured that all participants started the scene exploration initially, gazing in the same direction. Once the virtual room appeared, participants performed the task. After placing the target object in the final location, the trial was finished upon a button press, followed by the subsequent trial.

In Figure 4, an example of the scene view from the participant's perspective is shown.

#### 2.4.2. Training session and main experiment

During the experimental session, participants performed the training session followed by the main experiment. A short 3-to-5-min break was introduced between the two sessions, during which participants removed the VR headset and rested. At the beginning of each session, the eye tracker calibration procedure was performed. First, participants completed a training session. During 32 trials, participants executed the task. During the training session, in contrast to the main experiment, the set of target objects was compiled by images of objects typically found outdoors (e.g., a traffic lights, a park bench). However, the virtual rooms were identical to those in the main experiment (kitchens, bathrooms, offices, and empty rooms with shelves). The goal of the training session was for the participants to get familiar with the environments and become acquainted with the dynamics of the trials, picking up and transporting virtual objects and switching between the trials. After a break, participants proceeded to the main experiment. The procedure was identical to the training phase, except the target objects were context-dependent.

Each trial was unique in terms of the combination of a specific room type and a target object. In doing so, we intended to prevent learning of specific spatial configurations. The total amount of trials was then composed of seven unique target objects for each of three scene contexts designed in two variations and used in three experimental conditions (see Section 2.4.3). This resulted in a total of 126 trials for each participant. The order of trials was randomized, where all trials, including the training session, were performed within one 1-h appointment.

#### 2.4.3. Experimental conditions

To evaluate the impact of the scene context on the performance of the task, three experimental conditions were implemented. In the “congruent” condition, the target object matched the scene context (e.g., a toothbrush in a bathroom). In the “incongruent” condition, the object did not fit the scene context (e.g., a toothbrush in a kitchen). Specifically, the objects belonging to the remaining two contexts were randomly selected to form the incongruent condition. Finally, in the “empty” condition, no scene context was present.

Each room had a set of specific locations where the target object could appear. The possible locations were always on top of surfaces. The locations were determined by the proximity of each target object to its corresponding anchor (see Section 2.3.3). In the incongruent condition, the set of possible target locations was identical to that in the congruent condition, where throughout the trials each of the seven potential locations was occupied by one of the objects that did not belong to the context. Finally, in the empty condition, the configurations of the target objects, the distractors, and the virtual rooms were the same as those in the context-rich environments. This way, the possible spatial locations of the target objects were replicated in each of the three conditions, enabling the comparison across the conditions. In each trial, only one of the possible target objects was present.

Besides the target object, each room included a set of 10 distractors, which were located in specific positions in the context-rich and empty environments (Section 2.3.2).

### 2.5. Analysis

#### 2.5.1. Eye movements data pre-processing

To evaluate the task performance, first, the eye movement data were analyzed and fixations were detected. The eye movement data were recorded at a frequency 120 Hz. The gaze position data was accessed using a customized written Unity script utilizing the HTC SRanipal SDK package functions. The eye data processing flow was adapted from our previous work (Lukashova-Sanz and Wahl, 2021). In Table 1 the main, recorded variables are described. All variables were recorded for left and right eyes.

**Table 1:** Main eye- and head-movement-related raw variables recorded during the experiment.

**Variable**	**Units**	**Meaning**
Time stamp	An integer number	The time in ms at the moment of sample recording.
Eye data validity bit mask	An integer from 0 to 31	Represents the validity of the data. A value of 31 indicates the highest validity of the recorded data. This parameter is used to filter the raw data where the eye tracker lost the pupil, including filtering blinks.
Gaze normalized direction vector	A three-coordinates vector *(x, y, z)* with each coordinate ranging from −1 to 1	A gaze vector indicating the direction of gaze in the headset right-hand coordinate system.
Head rotation	A rotation quaternion *(x, y, z, w)* of head	A quaternion describing the rotation of the headset in Unity world coordinates.

To prepare the data for further processing, first, similar to (Imaoka et al., 2020), the raw data were filtered based on the eye data validity bitmask value, which represents the bits containing all validity for the current frame. After the filtering, only the data where the eye data validity bit mask had value 31 for both eyes Table 1, were selected. Doing so, the data where the eye tracker partly or completely lost the pupil (including blinks) was filtered out. Next, for subsequent fixation detection (see Section 2.5.2), the gaze position was calculated in spherical coordinates. In particular, the polar ϕ and azimuthal θ angles were computed using Equations (1) and (2). In Unity, the *z*-axis corresponds to the depth dimension.


(1) $$\begin{array}{r} \phi =\arctan\frac{x}{z}, \end{array}$$

(2) $$\begin{array}{r} \theta =\text{arctan2}\left(y,\sqrt{x^{2}+z^{2}}\right), \end{array}$$

where (*x, y, z*) are coordinates of normalized gaze directional vector in headset coordinates. Note that SRanipal returns the gaze direction vector in the right-handed coordinate system. To convert the coordinates in Unity world coordinate system, which is a left-hand coordinate system, the *x*-coordinate was multiplied by −1. To compute the gaze position in Unity world coordinate system, the gaze position in the headset coordinate system was multiplied by the head rotation quaternion.

#### 2.5.2. Fixation detection algorithm — I-VT

**Figure 5:**
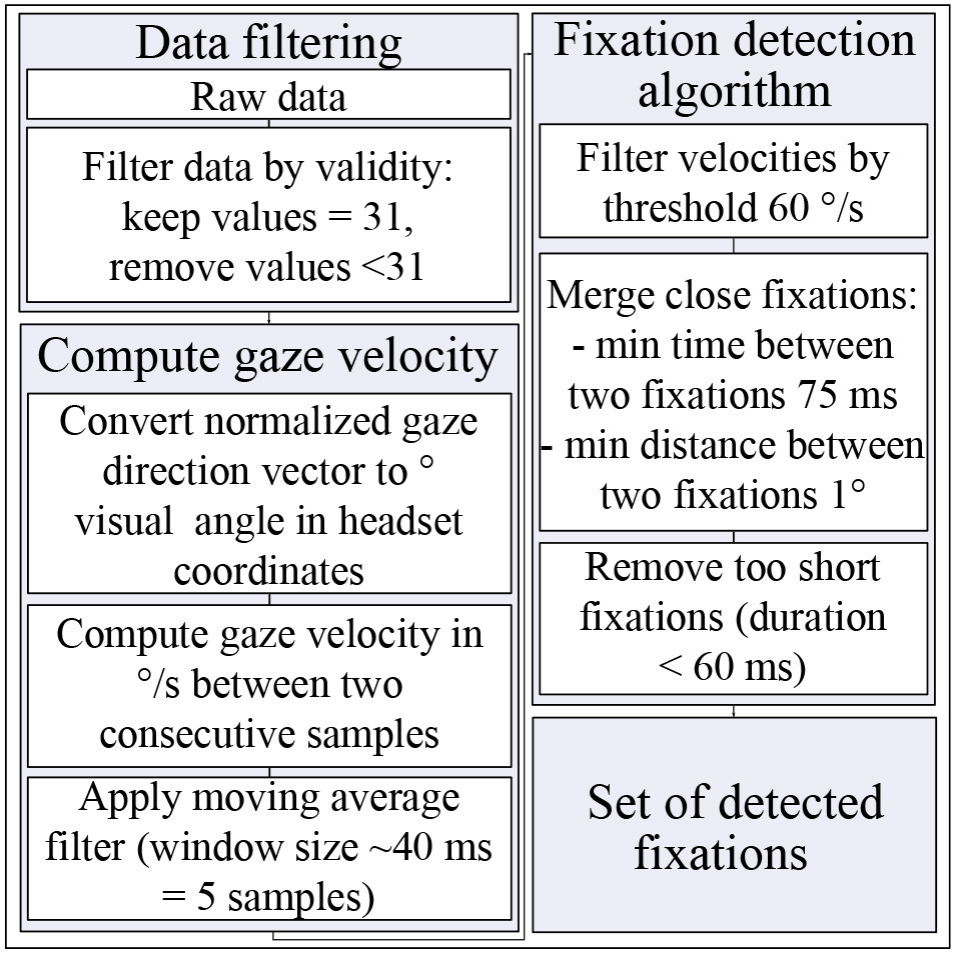
Eye movement data processing algorithm. For details, see Section 2.5.2.

Fixations were identified using *velocity threshold algorithm for fixation identification* (I-VT) (Salvucci and Goldberg, 2000). The algorithm was implemented following the description in (Kübler, 2020) and (Olsen, 2012). The gaze velocity *v* was computed in °/s between each two consecutive samples (Equation 3).


(3) $$\begin{array}{r} v=\frac{\sqrt{{\left(\phi_{i}-\phi_{i-1}\right)}^{2}+{\left(\theta_{i}-\theta_{i-1}\right)}^{2}}}{t_{i}-t_{i-1}}, \end{array}$$

where (ϕ_*i*_, θ_*i*_) and (ϕ_*i*−1_, θ_*i*−1_) are consecutive gaze positions in degrees of visual angle in headset coordinates, and *t*_*i*_ and *t*_*i*−1_ are respective time stamps. To reduce the noise level of the data, a running average filter was applied with the window size of five samples, which is ~ 40 ms. An eye movement was considered to be a fixation if the gaze velocity did not exceed a threshold of 60 °/s (Leube et al., 2017). Two fixations were merged in a single fixation if the time between them was under 75 ms (Komogortsev et al., 2010), and the angular distance was under 1° (Over et al., 2007; Komogortsev et al., 2010). Too short fixations with a duration under 60 ms were filtered out (Over et al., 2007; Komogortsev et al., 2010). In Figure 5 the eye movement data processing algorithm is summarized in a flow chart.

#### 2.5.3. Determining gaze position on a virtual object

To determine the gaze position on the virtual object, a hit point of the gaze ray and the 3D object collider were recorded for each frame. Using this approach, Unity returns a set of three coordinates of a specific spatial point on the collider surface which was crossed by the gaze ray. Furthermore, the name of the hit collider was continuously recorded. Doing so, it was tracked which object was gazed at, in which frame, and for how long.

#### 2.5.4. Task phases: search, reach, and transport

In each trial, the data was segmented into three phases: the search phase, the reach phase, and the transport phase.

The *search phase* is the period of time between the beginning of the trial and the first fixation on the target object. The first fixation on the object was defined as the first fixation during which the object collider was hit by the gaze ray. The *reach phase* was defined as the period of time between the first fixation on the target object and the moment of picking up the object, which is determined by the virtual hand attachment to the target object. Finally, the *transport phase* is the period of time starting from picking up the object until releasing it from the virtual hand when placing the object to the final location.

**Figure 6:**
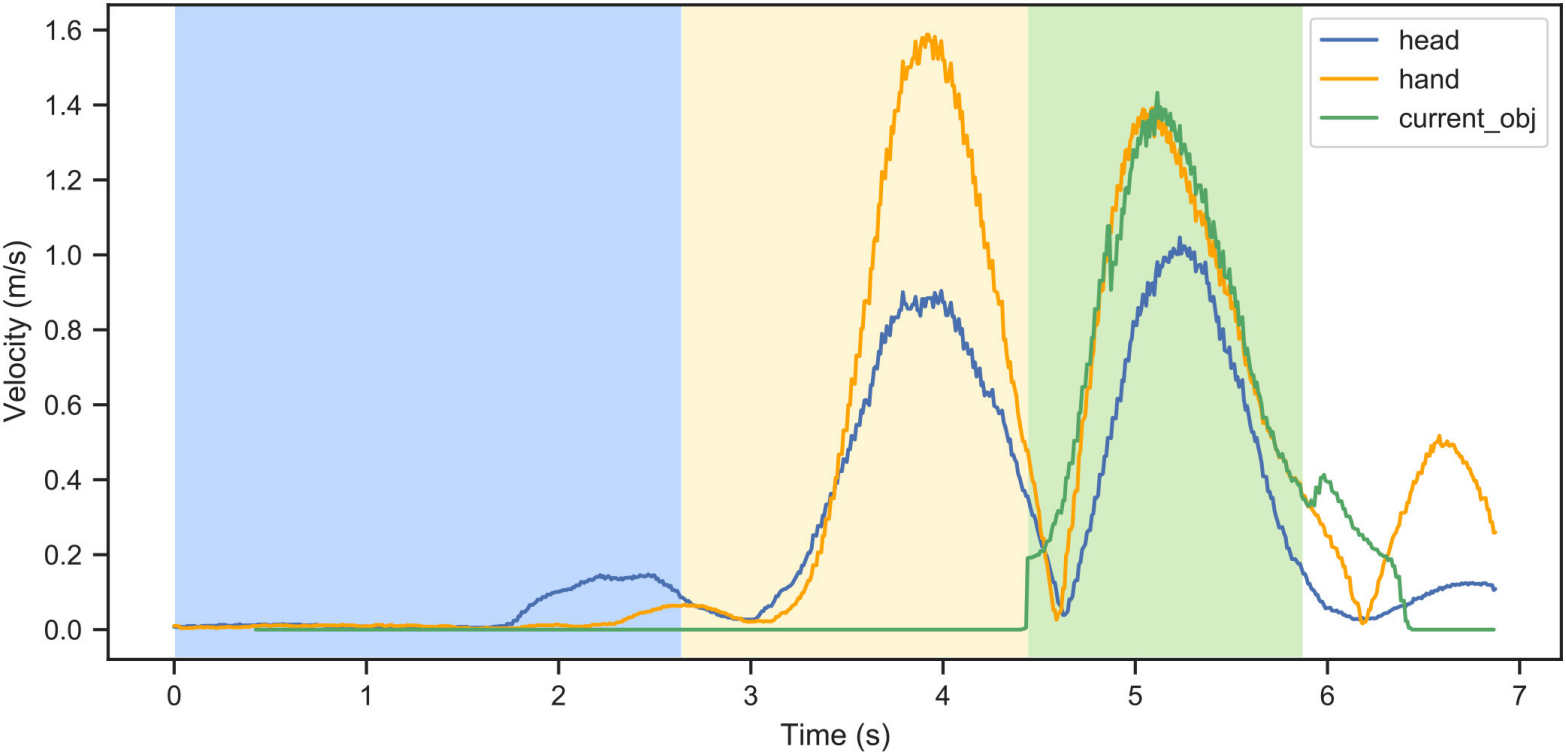
An example of velocities as a function of the trial duration for one participant in one trial: different curves represent the head, the hand, the target object, and the end target, respectively. The colored sectors correspond to the search (blue), reach (yellow), and transport (green) phases. In this example, the task was completed in approximately 7 s.

In Figure 6, an example of velocities for one participant in a single trial is shown. Different curves represent velocities of the head, the hand, the target object, and the end target location. The colored areas correspond to the search, reach, and transport phases.

#### 2.5.5. Behavioral metrics

As mentioned in Section 2.2.2, the statistical analysis was conducted using R version 3.6.1, in particular, package nlme (Pinheiro et al., 2022). As described in Section 2.4.2, a diverse range of the target objects was used for the experiment, where each trial was unique in terms of the combination of the target object and the corresponding scene. The models did not consider the specific objects or the scenes as an additional factor due to very limited amount of trials for each particular object and scene. The temporal data was normalized using log function, whereas the proportional data was not normalized. All models were fitted using REML (reduced maximum likelihood) method. Each dependent variable was fitted with a separate model. No multiple comparison correction was performed. More details on specific linear mixed models for each variable can be found in Supplementary material.

***Task duration***

To evaluate the impact of the scene context on task performance, first, the *task duration* was evaluated. The effect of the scene context was estimated by fitting a linear mixed model to the data, where *task duration* is a dependent variable, *condition* is a fixed effect, and *participant* is a random factor.

***Search, reach, and transport duration***

Next, the duration of different task phases: search, reach, and transport, was examined and compared across different experimental conditions. The impact of the scene context was estimated by fitting linear mixed models to the data, fitting a separate model for each of the metrics, where *search duration, reach duration*, and *transport duration* are dependent variables, *condition* is a fixed effect, and *participant* is a random factor.

For the search phase, it is important to mention, that the search time is naturally expected to depend on the initial gaze-object distance at the beginning of the trial. In such, if the target object was originally behind the participant upon the trial start, it is likely to take longer to find it, compared to when the object was initially in front of the participant. Therefore, for the search duration, the *initial gaze-object distance* was set as an additional fixed factor.

***Scene coverage***

Another parameter that is expected to be affected by the scene context is *scene coverage* – a common metric indicating the proportion of an area covered during the trial. To compute the scene coverage in each trial, first, a 2D histogram of gaze points was plotted for the scene where the whole span of 360 °va and 180 °va in horizontal and vertical directions, respectively, was considered. The size of the histogram bins was set to 2 °va which approximately corresponds to the eye tracker accuracy. The histogram was then transformed into a binary image, with black pixels representing the area in which some gaze points fell. Finally, the scene coverage was computed as the proportion of the black pixels to the total amount of pixels of the scene. Similarly to the task and search duration, the impact of scene context was estimated by fitting the linear mixed models to the data, where *scene coverage* is the dependent variable, *condition* is a fixed effect, and *participant* is a random factor.

***Proportion of gaze on target object and anchor***

The *proportion of gaze on target object* was defined as the proportion of the number of frames gazing on the target object out of the total amount of recorded frames of the trial. In line with other metrics, this parameter indicated whether the scene context facilitated the task performance, which would be implicitly demonstrated by a larger proportion of gaze on the target object.

The *proportion of gaze on the anchor* was computed similarly as the proportion of the number of frames gazing on the anchor relative to the total number of frames in the trial. Note, that this metric was computed only for the context-rich conditions as in the empty condition no context and, therefore, no anchors were present. Furthermore, in the congruent condition, the anchor was relevant to the target object, whereas in the incongruent condition, even though in the same spatial configuration, semantically it was irrelevant. This metric enabled an implicit evaluation of the context facilitation, namely, a larger proportion of gaze on the anchor would demonstrate the importance of the relevant anchor for the task performance.

For both metrics, the impact of the scene context was evaluated by fitting the linear mixed models to the data, where a separate model was fitted to each metric. In the model, the *proportion of gaze on object and anchor* are dependent variables, *condition* is a fixed effect, and *participant* is a random factor. Note, that due to the realistic nature of the scenes, the anchors varied in size as well as in their relative position to the target object (see Section 4.4).

***Anchor-object transition***

Finally, the *anchor-object transition* was evaluated. The anchor-object transition is the time between the first fixation on the anchor and the first fixation on the target object. A positive value corresponds to when the anchor was fixated before the target object, whereas negative values show that the target object was fixated before the anchor. A shorter anchor-object transition would indicate the importance of the anchor. Note, that this metric was computed only for the context-rich conditions, as in the empty condition, no anchor was present. A linear mixed model was fitted to the data with *anchor-object transition* as dependent variable, *condition* as fixed effect, and *participant* as random factor.

## 3. Results

In this section, we report the mean values of each variable of interest together with their standard deviation. Using the nlme package (Pinheiro et al., 2022), the model output summary returns the fixed effects estimates, their approximate standard errors, the denominator degrees of freedom, the ratios between the estimates and their standard errors, and the associated *p*-values from a t-distribution. In the present work, the significance of the scene context effect on various dependent variables was evaluated based on the computed *p*-values returned in the output of the respective models. The output of each model can be found in the Supplementary material.

During a few trials, the connection between Unity and the headset was lost. After the data curing, for each participant, a total of maximum four trials was excluded from further analysis.

Most of the variables are visualized as bar plots with an overlay of individual subject values. The error bars correspond to the 95% confidence intervals. The default Seaborn (Waskom et al., 2017) setting was used to compute the confidence intervals, namely, through bootstrapping by sampling 1000 samples uniformly with replacement from the original data.

### 3.1. Task duration

**Figure 7:**
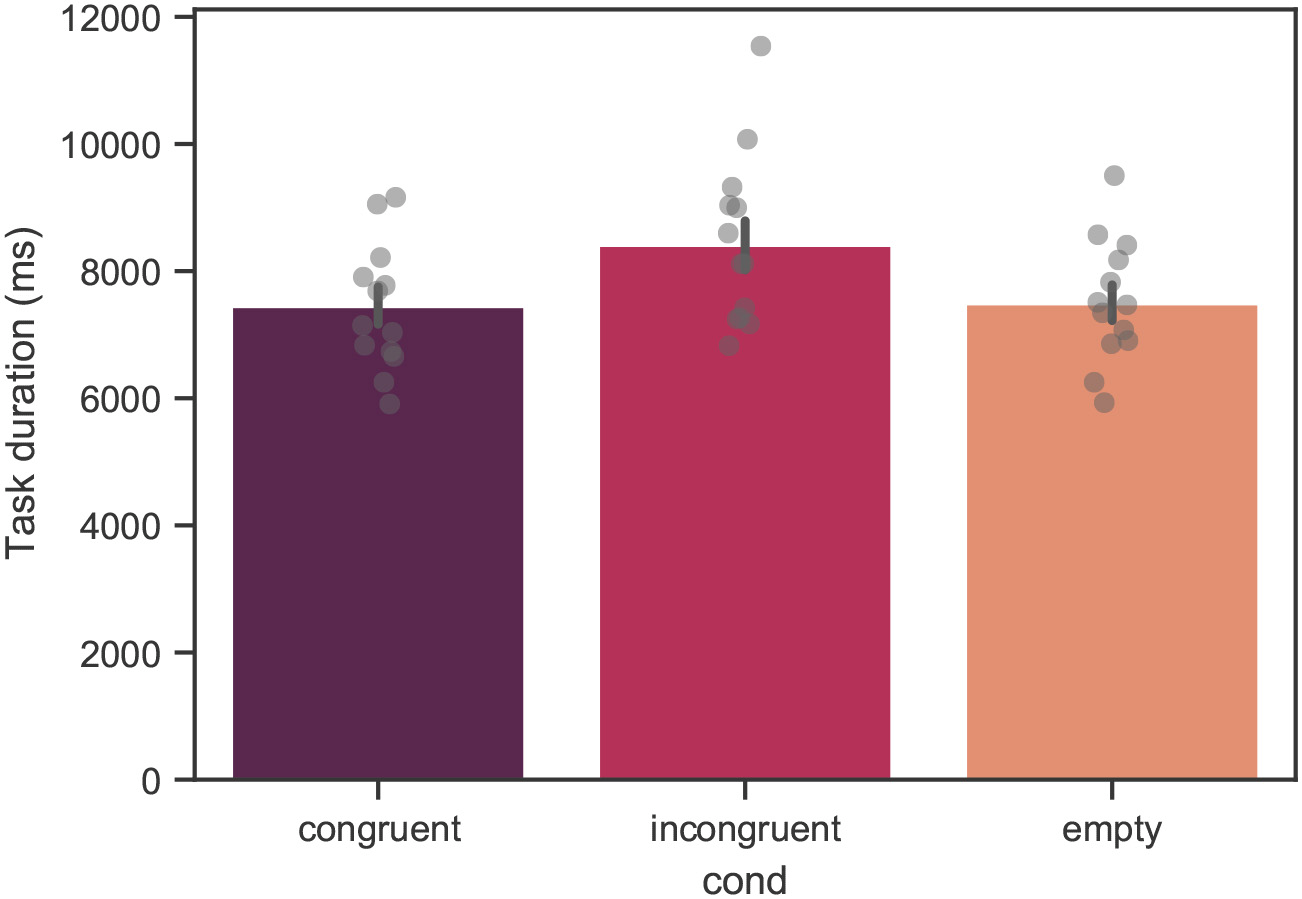
Mean task duration across all trials for all measured participants. Each bar corresponds to a separate experimental condition. The error bars indicate confidence intervals of 95% computed using bootstrapping (see main text). The individual points correspond to the mean value for each individual subject. The difference between the congruent and incongruent conditions was significant with *p* < 0.001. The difference between the congruent and empty conditions was not significant.

In Figure 7, the mean task duration estimated across all participants is shown. The mean values of the task duration were 7433 ± 2820, 8395,± 3822, and 7487 ± 2767*ms* for the congruent, incongruent, and empty conditions, respectively. From linear mixed model analysis, over the course of all trials, a significant effect of condition was found with *p* < 0.001. The difference between the congruent and empty conditions was not significant.

### 3.2. Search, reach, and transport duration

**Figure 8:**
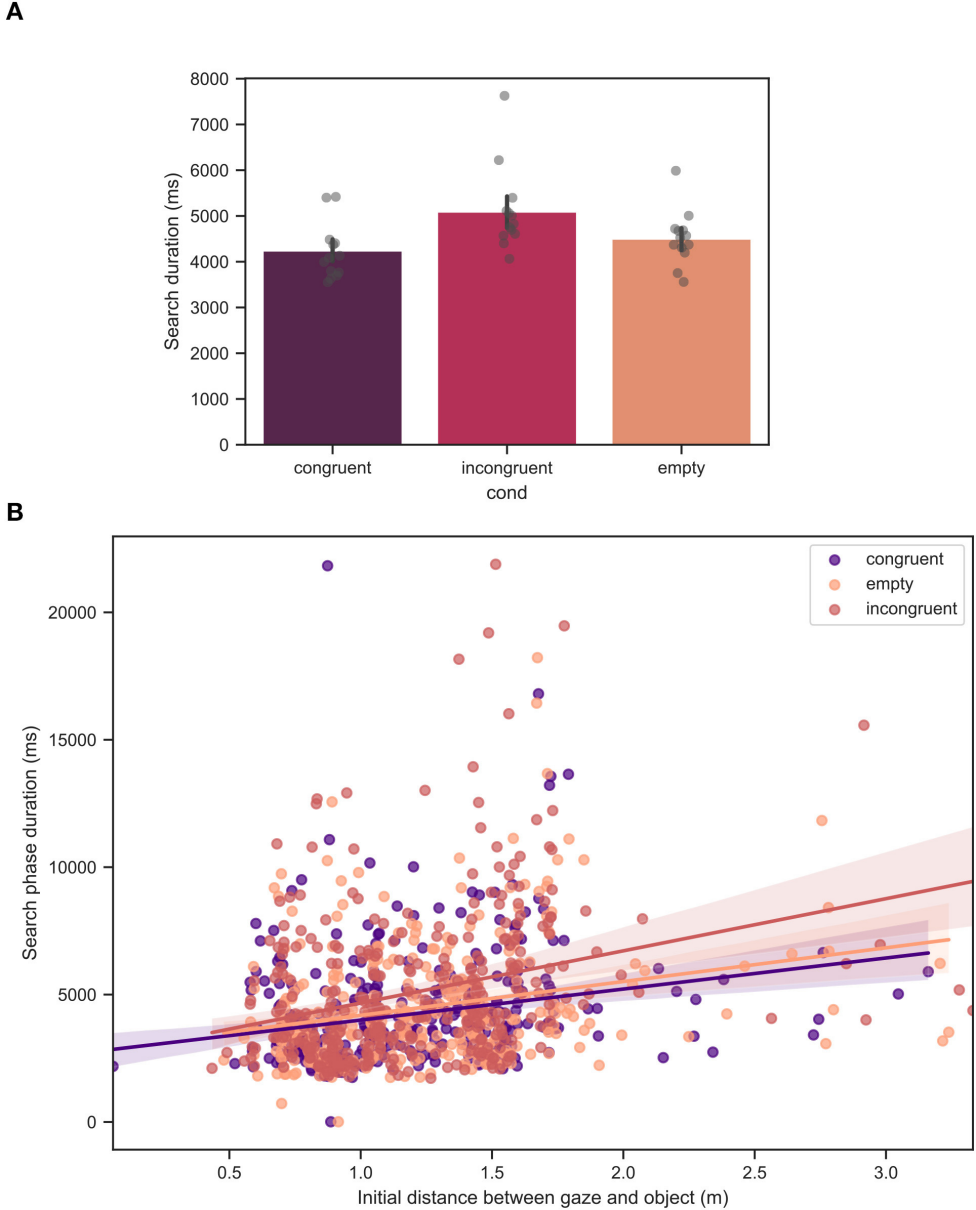
**(A)** Mean search duration across all trials for all measured participants. Each bar corresponds to a separate experimental condition. The error bars indicate confidence intervals of 95% computed using bootstrapping (see main text). The individual points correspond to the mean value for each individual participant. The difference between the congruent and incongruent conditions was significant with *p* < 0.001. The difference between the congruent and empty conditions was not significant. **(B)** Mean search duration as a function of the initial gaze-object distance, defined as the distance between the looked at point and the target fixation at the beginning of the trial. The straight lines are linear fits to the data. The effect of the initial gaze-object distance was significant with *p* < 0.001.

In Figure 8A, the mean search phase duration across all participants is shown. The mean values of the search duration were 4232 ± 2266, 5073 ± 3040, and 4499 ± 2377*ms* for the congruent, incongruent, and empty conditions, respectively. From linear mixed model analysis, over the course of all trials, a significant effect of condition was found with *p* < 0.001. Figure 8B demonstrates the search duration as a function of the initial gaze-object distance. As expected, there was found a significant effect of the initial distance, with *p* < 0.001 which is indicated by a positive slope of the linear fit for all the conditions. The difference between the congruent and empty conditions was not significant.

**Figure 9:**
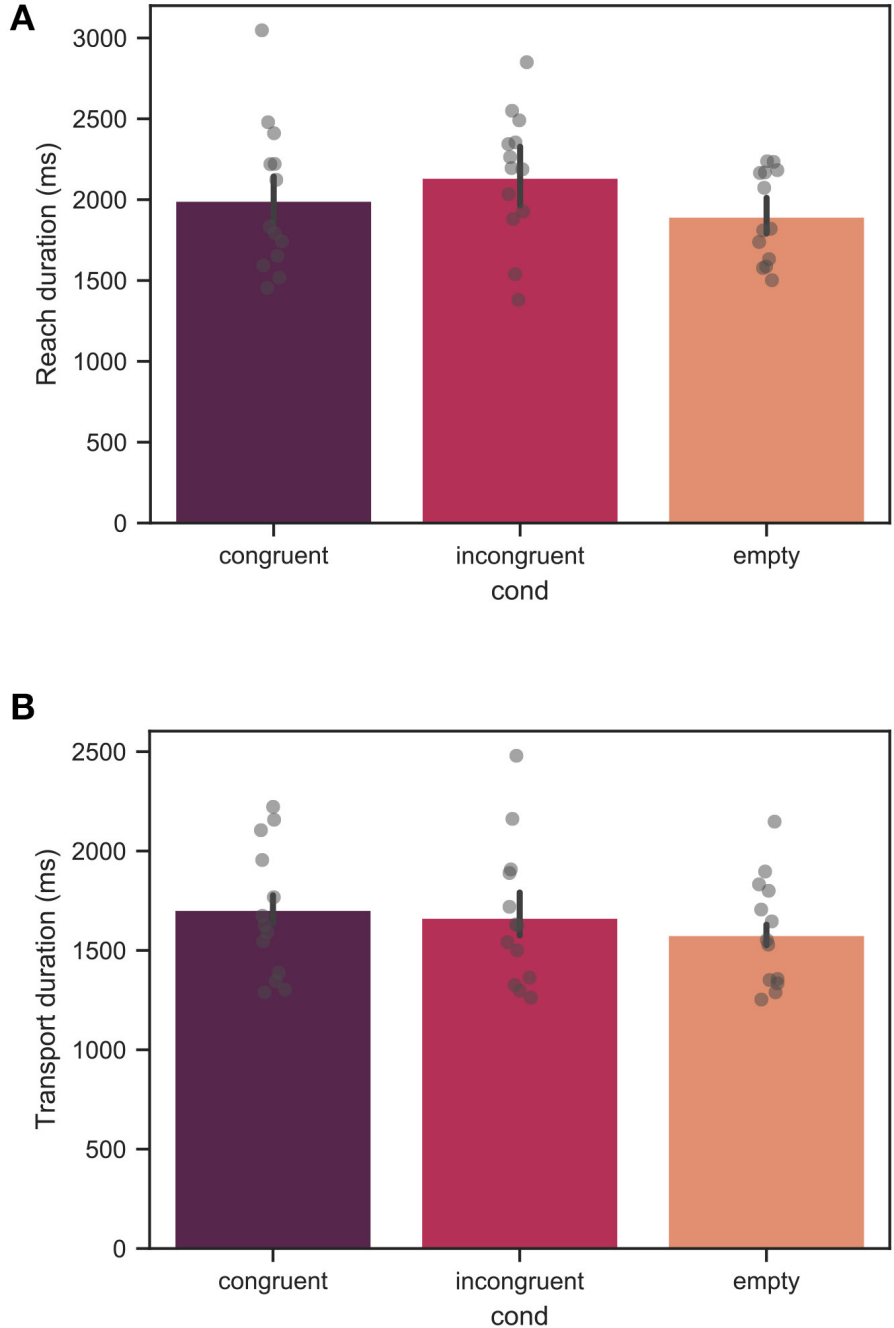
Mean duration of the **(A)** reach phase, and **(B)** transport phase across all trials for all measured participants. Each bar corresponds to a separate experimental condition. The error bars indicate confidence intervals of 95% computed using bootstrapping (see main text). The individual points correspond to the mean value for each individual participant. For the reach phase, the effect of condition was not significant. For the transport phase, the difference between the congruent and empty conditions was significant with *p* < 0.01, whereas no significant difference was found between the congruent and incongruent conditions.

Figures 9A,B show the reach and transport duration across all trials for all measured participants. For the reach phase, no significant effect of the context condition was found. For the transport phase, the duration in the empty condition was significantly shorter than in the congruent condition with *p* < 0.01. The mean values for the reach phase duration were 1992 ± 1367, 2140 ± 1744, and 1895 ± 1046*ms* for the congruent, incongruent, and empty conditions, respectively. The mean values for the transport phase duration were 1753 ± 657, 1666 ± 1057, and 1578 ± 475*ms* for the congruent, incongruent, and empty conditions, respectively.

### 3.3. Scene coverage

**Figure 10:**
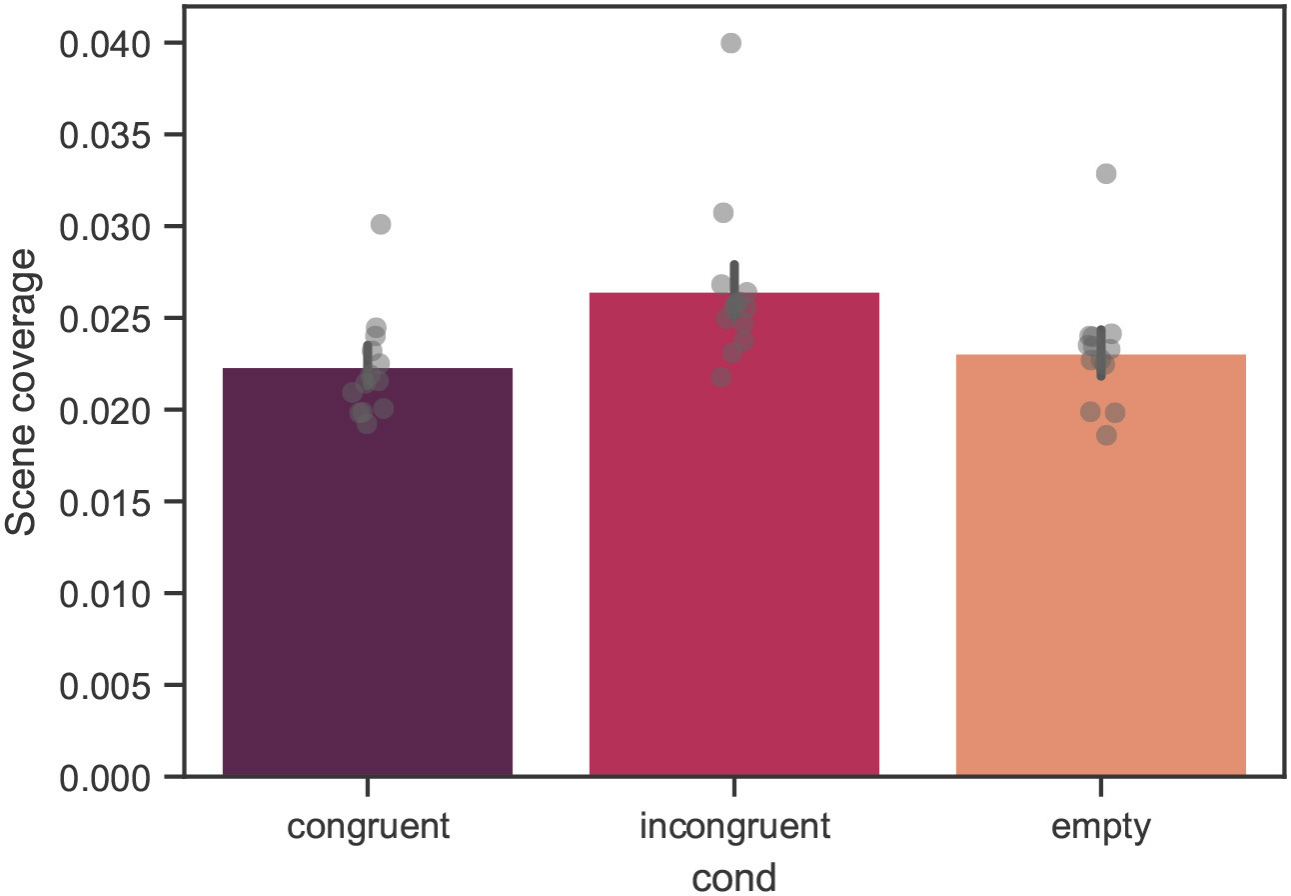
Mean scene coverage across all trials for all measured participants. The variable is computed as a proportion, thus, ranges between 0 and 1. Each bar corresponds to a separate experimental condition. The error bars indicate confidence intervals of 95% computed using bootstrapping (see main text). The individual points correspond to the mean value for each individual participant. The difference between the congruent and incongruent conditions was significant with *p* < 0.001. The difference between the congruent and empty conditions was not significant.

In Figure 10, the mean scene coverage across all trials for all participants is shown. The mean values of the scene coverage were 0.022 ± 0.010, 0.026 ± 0.014, and 0.023 ± 0.012 for the congruent, incongruent, and empty conditions, respectively. From linear mixed model analysis, over the course of all trials, a significant effect of condition was found with *p* < 0.001. The difference between the congruent and empty conditions was not significant.

### 3.4. Proportion of gaze on target object and anchor

**Figure 11:**
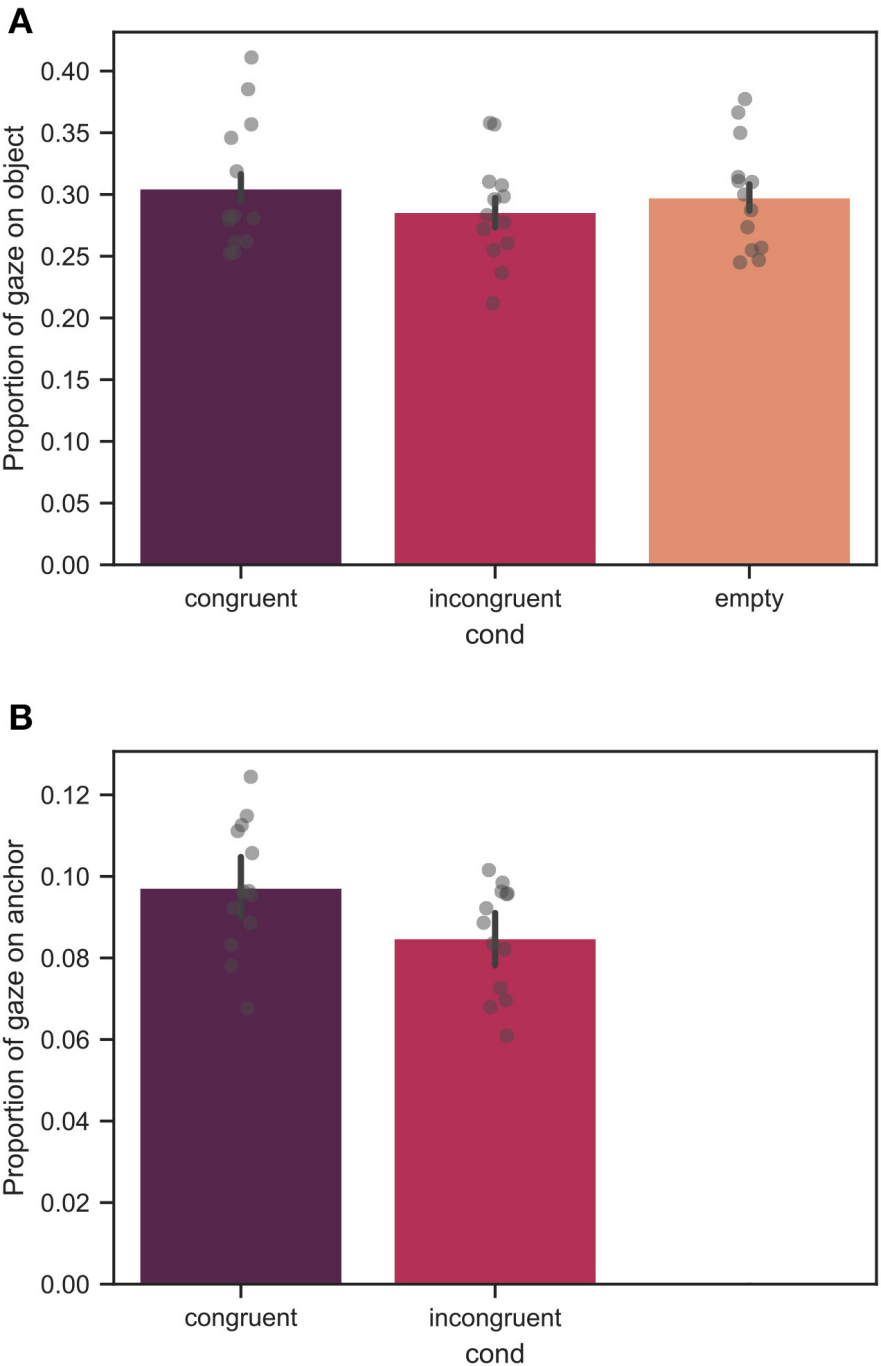
The proportion of gazing on the **(A)** target object and **(B)** relevant anchor across all trials, averaged over all measured participants. Each bar corresponds to a separate experimental condition. The error bars indicate confidence intervals of 95% computed using bootstrapping (see main text). The individual points correspond to the mean value for each individual participant. The difference in **(A)** between the congruent and incongruent conditions was significant with *p* < 0.01, whereas no significant difference was found between the congruent and empty conditions. Note, that in **(B)** the proportion of gazing on the relevant anchor is shown only for the context-rich conditions as in the empty condition no anchor was present. There was a significant difference for the proportion of gazing on the relevant anchor with *p* < 0.05.

In Figure 11, the proportion of gazing on the target object and the relevant anchor averaged over all participants is shown. Note, that Figure 11B demonstrates data only for the two context-rich conditions as in the empty condition no context and, thus, no anchor was present. The mean values of the proportion of gazing on the target object were 0.31 ± 0.11, 0.29 ± 0.11, and 0.30 ± 0.10 for the congruent, incongruent, and empty conditions, respectively. The mean values of the proportion of gazing on the relevant anchor were 0.10 ± 0.07, and 0.09 ± 0.06 for the congruent, and incongruent conditions, respectively.

From linear mixed model analysis, over the course of all trials, a significant effect of condition for the proportion of gazing on the target object with a significant difference between the congruent and incongruent conditions with *p* < 0.01 was found. The difference between the congruent and empty conditions was not significant. Moreover, a significant effect of the context condition was found for the proportion of gazing on the relevant anchor with *p* < 0.05.

### 3.5. Anchor-object transition

**Figure 12:**
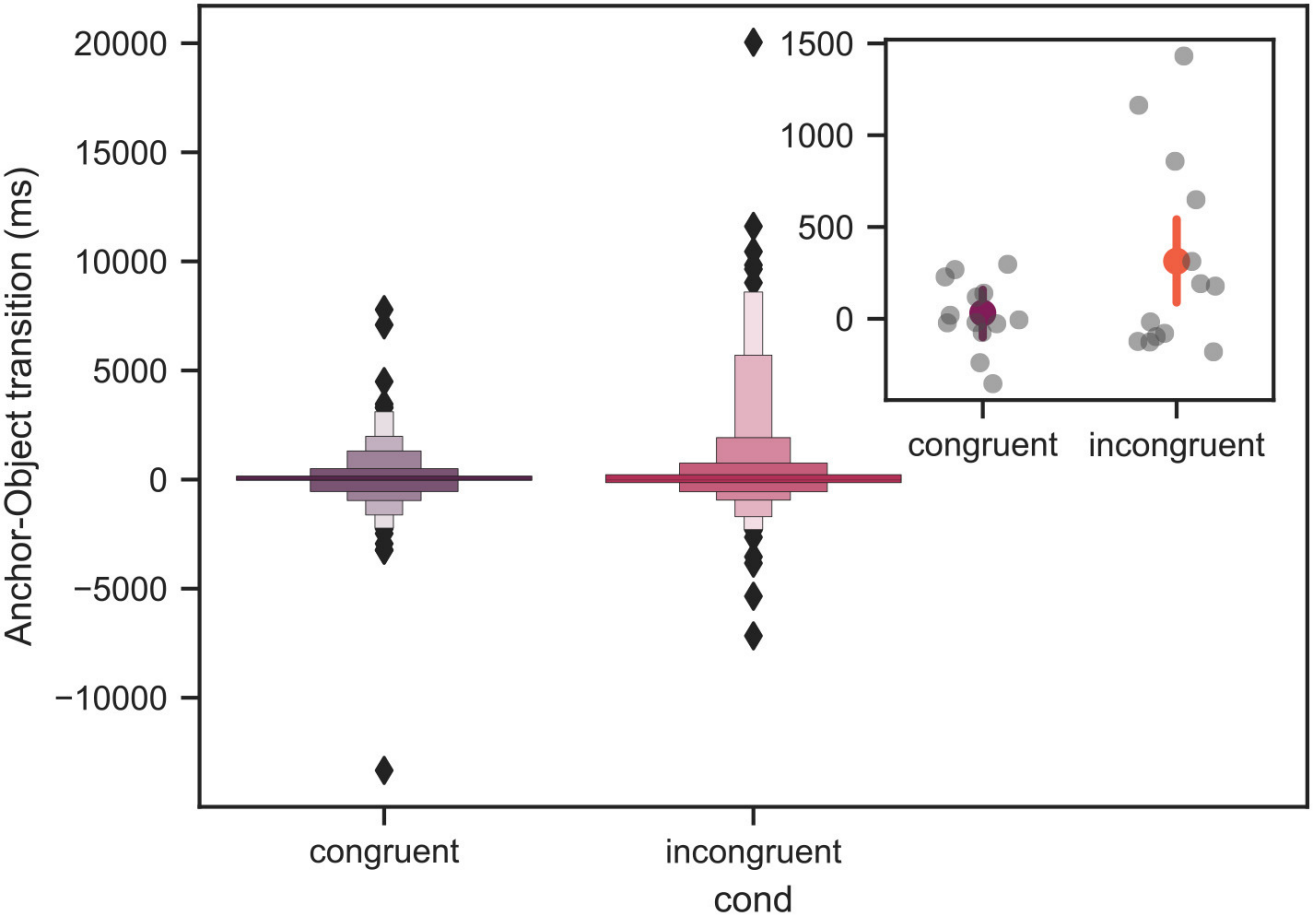
Mean anchor-object transition across all trials for all measured participants represented by boxen plots. The black diamond markers correspond to the outliers. For more details on the boxen plot structure see main text. The inset in the upper right location demonstrates the mean values with the respective 95% confidence intervals computed using bootstrapping (see main text). The gray individual points in the inset plot correspond to the mean value for each individual subject. The axes labels of the inset plot are identical to those of the main plot. There was a significant difference between the congruent and incongruent conditions with *p* < 0.05.

Figure 12 shows the anchor-object transition represented by boxen (letter-value) plots. For a large data set, this advanced boxplot type offers an advantage for visualizing the data distribution as it prevents a visual overload by the outliers. For more details on the boxen plots see Seaborn documentation (Waskom et al., 2017). The mean values across all participants with the respective confidence intervals, as well as the individual average values for each participant are shown in the inset plot in Figure 12.

Note, that the positive values indicate that the object was gazed at after the anchor, whereas the negative values correspond to the case when the object was gazed at before the anchor. Furthermore, the data is shown only for the context-rich conditions, as in the empty condition no anchor was present. From linear mixed model analysis, over the course of all trials, a significant difference between the congruent and incongruent conditions was found with *p* < 0.05.

## 4. Discussion

Only a few studies looked into the scene context impact on human behavior in an interactive task until now. The present study investigated how the scene context affects human object manipulation in a pick-and-place task in a realistic scenario. This study examined whether object manipulation in an isolated setting differs once the object of interest is brought into a scene context. Using a psychophysics approach implemented in a VR environment, we evaluated behavior during three phases: the search, the reaching and picking of the object, and then transporting it to a predefined final location. Specifically, the performance was evaluated in three different conditions: when the object matched the scene context, when it did not fit the context, and when no context was present.

Overall, the experimental paradigm captured well an interactive task in a realistic 3D environment. The possibility of freely moving head and gaze, as well as unconfined hand movement, brought the controlled experimental setting closer to a real-life scenario compared to traditional screen-based paradigms.

### 4.1. Task duration, search duration, scene coverage

The significant increase of almost a second, which was found for the task duration in the incongruent condition compared to the congruent condition, shows general facilitation of the task performance by the scene context, which is also apparent from previous studies (Biederman et al., 1982; Võ and Wolfe, 2013). In particular, the performance improvement in the present study was caused mainly by the search duration difference, indicated by a significantly longer search time for the incongruent condition. These results are in line with the previous research on the impact of the scene context on visual search (Boettcher et al., 2018; Võ, 2021). Likewise, the eye movement data examination showed a corresponding increase in the proportion of the scene covered by the gaze for the incongruent condition, indicating that when the object did not match the scene context, participants had to gaze around more compared to when the object semantically matched the scene. One explanation of this behavior has been previously proposed in the literature (Biederman et al., 1982; Bar, 2004, 2009). In particular, it was suggested that context (also referred to as the scene grammar) facilitation of individual object recognition in a scene originates in the generation of specific predictions by the observer, which can be later used to find the object. Such, when the object recognition cannot be rapidly resolved based solely on the physical features of the target object, contextual information can contribute more to the efficient recognition of that object than its physical attributes (Bar, 2004, 2009).

Notably, no significant differences were found for the task duration, the search duration, or the scene coverage when comparing the congruent and empty conditions. These results indicate that although there is substantially more visual content in a context-rich environment, it does not seem to distract the user from the task performance. And instead, it enables an efficient search process. Nonetheless, when the target object is placed in the same scenes and the same spatial configurations but does not match the context, the performance decreases, underlining the role of the scene context. In line with previous research, these findings suggest that in the context-poor “empty” environment, the participants based their search primarily on the physical attributes of the target. When the same target was placed in the incongruent scene context, that is, the contextual information was non-informative, a substantially larger number of visual stimuli served as a distractor for the searcher and led to longer search times. In contrast, when the contextual information was relevant, it compensated for a large number of visual stimuli. It is suggested, that the context maintained the search time comparable to that in the “empty” scenario.

It is worth mentioning that the present interpretation of the results is based on the assumption that the search in the empty condition is relatively complex. During the study development, as mentioned in Section 2.3.2, the number of distractors for all scenes was selected rather large to ensure a considerable search time even for the empty condition, here, at least 4 s on average. However, no detailed analysis of the search complexity in the empty condition at a given number of distractors was performed. To strengthen the present results and their interpretation, in future studies, it is recommended to deliberately analyze the search complexity in the empty condition, for example, through systematic modulation of the number of distractors and evaluation of the respective search times.

### 4.2. Reach and transport duration

Until now, few studies attempted to describe the effect of the scene context in an interactive task rather than a search display. In one recent study, participants were asked to construct environments from a set of various available virtual objects, either according to the contextual scene expectations of the observer or against them (Draschkow and Võ, 2017). Among other findings, the authors demonstrated that participants held objects for a longer time in the context-incongruent condition. In the present study, we similarly evaluated whether the scene context affected the transport duration of the target object. In contrast to (Draschkow and Võ, 2017), in the present study, the final location was predefined and kept constant throughout all trials. Therefore, participants did not need additional time to decide where to put the object. Nonetheless, it took participants less time to transport the object to its final location in the empty condition. Further analysis did not reveal any differences in the number of gaze points on the target object during the transport phase across the conditions. We, therefore, speculate that since the final location was predefined and the target object was not looked at more in any of the conditions during its transport, no additional processing was necessary. However, in context-rich environments, participants had to overcome more obstacles when transporting the target object compared to empty rooms with shelves, thus, leading to a slightly longer transport duration. Beyond the scope of the current work, further studies should address whether the uncertainty of the final location would introduce a variation in transport duration in different scene context conditions, as well as systematically explore the impact of the obstacles.

Considering the reach phase duration, previous studies have demonstrated motor inhibition when approaching dangerous objects due to the emergence of aversive affordances (Mustile et al., 2021). Furthermore, from visual search research, it is known that violations of one's scene grammar lead to longer and more fixations on the critical objects, which is typically attributed to more extended processing of those objects (Henderson et al., 1999; Cornelissen and Võ, 2017; Draschkow and Võ, 2017). In the present study, no significant differences in the reach duration were revealed across the conditions, meaning no effect of the scene context was found on the reach duration. It is suggested that if the exact target object is known before the trial start, it is still harder to find it in a semantically not matching context. However, once it is located, no extra processing is necessary. Therefore, no significant elongation of the reach phase emerged in the incongruent condition. Further studies are required to evaluate the impact of the scene context on the reach phase when the target object is unfamiliar to the observer before the task.

### 4.3. Target object and anchor

A small but significant difference in the gaze proportion on the target object correlated with the notion of task facilitation by the scene context. As expected, participants spent less time and effort to find the object in the congruent condition, resulting in proportionally more gazing at the object. More interesting, however, was the significant difference in gazing on the relevant anchor across the conditions. In particular, the anchor was gazed on more often when it could potentially be helpful to perform the task, meaning, in the congruent condition. The role of the anchors became more apparent from the recent studies (Boettcher et al., 2018; Võ, 2021). In natural contexts, people seem to be able to exploit the knowledge about the scene configurations when looking for an object (Võ and Wolfe, 2013; Draschkow and Võ, 2017). Furthermore, people tend to rely on a rather global context than local information, and, thus, the larger scene-typical objects—anchors—appear to have more influence on the search facilitation. The present study results confirm this notion, where the anchor in the incongruent condition appeared to be less supportive of the task performance in contrast to the congruent condition.

Considering the previously suggested scene processing (Võ, 2021) where the small objects are located after larger global objects, we generally expected the anchors to be fixated before the target objects in most of the trials. In contrast, the distribution of the anchor-object transition did not confirm our expectations. From literature, it is known that the search strategy can be composed of several processes, including feature guidance as well as scene guidance (Võ and Wolfe, 2013). In the current study, it is speculated that participants utilized scene guidance to facilitate the search process throughout the trials, which is reflected in better task performance. However, the unique appearance of the target objects and distractors in a given scene defined a set of specific features which could enforce the feature guidance (grayscale same-size cubes). Therefore, the anchors were not always fixated before the target object. Nonetheless, the anchor-object distribution appeared to be significantly narrower in the congruent condition, which underlines the role of the anchors even if it was fixated after the target object was already located. Further studies are necessary to investigate the dynamics of the mutual object and anchor fixations. Moreover, in the future studies it would be interesting to further address the dynamic nature of fixating the target object and the anchor throughout the search.

### 4.4. Limitations

Although the present work captured the effect of the scene context on the performance in an interactive pick-and-place task, it is important to comment on its limitations. First, even though there are recent attempts to formally define what anchors are (Draschkow and Võ, 2017), to the best of our knowledge, there is no existing database. Therefore, the anchors used in this study were selected intuitively based on a common understanding of the typical scenes. This, in turn, could inflate the individual differences in perceiving the intended anchors as such and reduce the effect of the scene context on the task performance. Furthermore, due to the natural scene contexts, it was not possible to design the anchors of uniform size as well as set the target object to the same position relative to the anchors. Thus, although the target objects were always in the proximity of the anchors, in some configurations, they were next to the anchors, whereas, in others, they were directly on top of them. This could, for example, influence the anchor-object transition. Another challenge for the paradigm design was variability in the similarities between the target objects and the distractors. As such, a toothbrush is more likely to be at first confused with a fork than with a soap dispenser due to the shape similarity, which would possibly increase the total search time of the target object. It is not a straightforward task to avoid this limitation due to the realistic nature of the objects and the scenes. Nonetheless, it could be advantageous to do a more systematic generation of the target objects and distractors sets in the future. Furthermore, in the present study, the effect of specific objects and scenes was not tested due to a very limited number of trials per object and scene. In future studies, it would be interesting to address how specific objects and scenes impact the behavior in a pick-and-place task. Finally, in the present study, grasping was implemented using the VR controller. While VR offers an excellent opportunity to simulate realistic scenarios in a lab environment and accurately track hand motion, a more natural solution would be to implement the grasping using only the participant's hand without a controller. This, however, significantly increases the complexity of the setup when it comes to reliable controller-free object manipulation. When manipulating objects in VR, some recent studies demonstrated a strong sense of ownership when the virtual hand is represented by a hand-like object which we also used in the present study. Nonetheless, the direct transformability of the current study results in a real-world grasping scenario should be a focus of future studies. For example, it would be interesting to compare the dynamics of a pick-and-place task in the real scenario and its replica in a virtual environment.

## 5. Conclusion

To conclude, this work evaluated the impact of the scene context on the performance of an interactive task, precisely, the pick-and-place task where the object had to be found, picked, and transported to a predefined location. In line with visual search literature, we found a disadvantage in search time when the object does not belong to the scene context compared to the context-congruent condition. When comparing the congruent and no-context conditions, the search performance was similar. This finding supports the notion that when the object fits the scene, the other objects and the context-rich environment itself seem to not introduce an additional distraction for the searcher and keep the search efficient.

The reach phase duration was not affected by the scene context. A small difference was found in the transport phase duration between the empty condition and both context-rich conditions. However, as discussed in Section 4.2, the elongation seems to be originating from the need to overcome some obstacles in the context-rich environments and not due to additional processing. Although this suggestion requires further systematic testing, at least in the present configuration where the final location for the object was known, the semantic congruency of the object and the scene context does not seem to affect the interactive phases of the pick-and-place task. This strengthens the validity of transferring eye and hand movement knowledge in a grasping task performed in an isolated setting to a realistic scenario within a context-rich environment.

The present study contributes to a better understanding of the dynamics of the pick-and-place task once the target object is placed in a realistic context-rich scene. Keeping the possible applications in mind, the findings of this work provide insights into the potential development of supporting intention predicting systems. In particular, the information about the object's semantic congruency with the scene context could potentially be used as an additional input parameter to train and calibrate future assistive algorithms for the support system. On a broader scope, the findings of the present study can be relevant for designing intention prediction-based assistive systems helping, e.g., visually impaired with intelligent tunable lenses, or to control prosthetics like robotic arms, wheelchairs, or exoskeletons.

## Data availability statement

The datasets presented in this study can be found in online repositories. The names of the repository/repositories and accession number(s) can be found below: osf.io/grm45.

## Ethics statement

The studies involving human participants were reviewed and approved by Faculty of Medicine at the University of Tübingen with a corresponding ethical approval identification code 986/2020BO2. The patients/participants provided their written informed consent to participate in this study.

## Author contributions

OL-S, RA, and SW developed the research idea, designed the experiment, edited and finally approved the manuscript. OL-S programmed the experiment, collected the data, conducted the analysis, and wrote the first draft of the manuscript. All authors contributed to the article and approved the submitted version.

## Funding

The present research was supported by the funding from the European Union's Horizon 2020 research and innovation program under Grant agreement No. 951910, and by the German Research Foundation (DFG): SFB 1233, Robust Vision: Inference Principles and Neural Mechanisms, TP TRA, project number: 276693517.

## Conflict of interest

We declare that OL-S and SW are scientists at the University of Tübingen and employees of Carl Zeiss Vision International GmbH, as detailed in the affiliations. The remaining author declares that the research was conducted in the absence of any commercial or financial relationships that could be construed as a potential conflict of interest.

## Publisher's note

All claims expressed in this article are solely those of the authors and do not necessarily represent those of their affiliated organizations, or those of the publisher, the editors and the reviewers. Any product that may be evaluated in this article, or claim that may be made by its manufacturer, is not guaranteed or endorsed by the publisher.
