# Supplementary material for: Context matters during pick-and-place in VR: Impact on search and transport phases
Source: Front Psychol. 2022 Sep 8;13:881269. doi: 10.3389/fpsyg.2022.881269 (PMC9493493; doi:10.3389/fpsyg.2022.881269)
Supplement: Supplementary file 1 [file Data_Sheet_1.pdf]

Supplemetary information

S1 VR scenes

In Figure S1 – Figure S2 the complete set of VR scenes used in the experiment is demonstrated. Three different typical contexts were selected: kitchen, bathroom, and office. Each context was designed in two variations. Furthermore, for each context-rich scene, an empty scene was created where the surfaces were substituted by shelves. Thus, a total of 12 realistic VR scenes were designed for the experiment.

S2 The target objects, distractors, and anchors

In Table S1 the pairs of the target objects and their corresponding anchors are shown. Furthermore, the distractors for each of the contexts are demonstrated. All objects were created by projecting an image on the cube facets. All images were downloaded from open sources with CC0 license, and thereafter, cropped and converted to the grey scale (for details see the main text).

S3 Free 3D assets used for the experiment

All scenes were created using freely available 3D assets with CC0 or CC BY license at the moment of download. In Table S2 all downloaded assets are referenced.

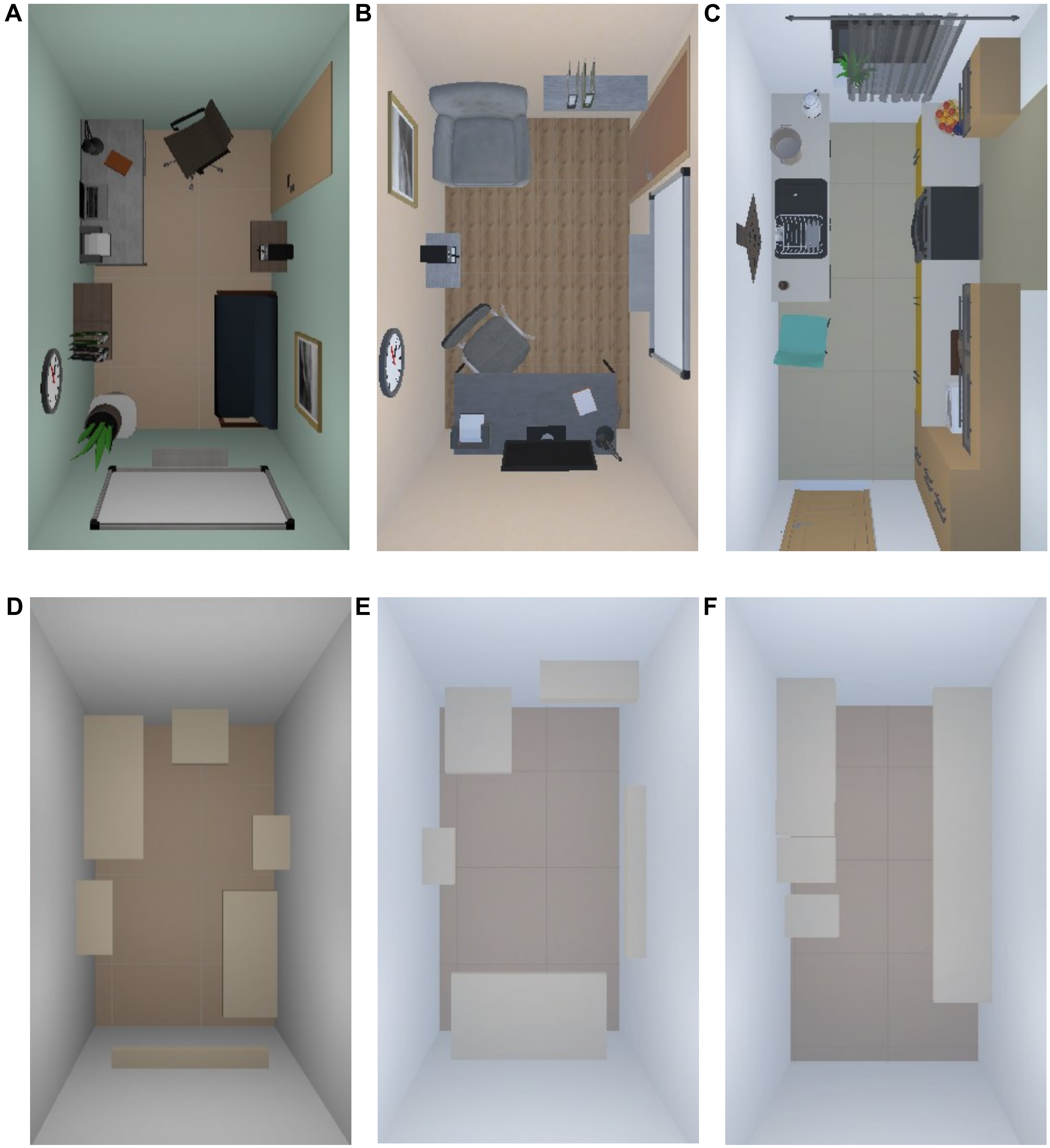

Figure S1: (**A**, **B**, **C**) the first three of the context-rich VR scenes from a total of six scenes, and (**D**, **E**, **F**) their empty matches. All scenes were designed using freely available 3D assets with CC0 or CC BY license (see Section S3).

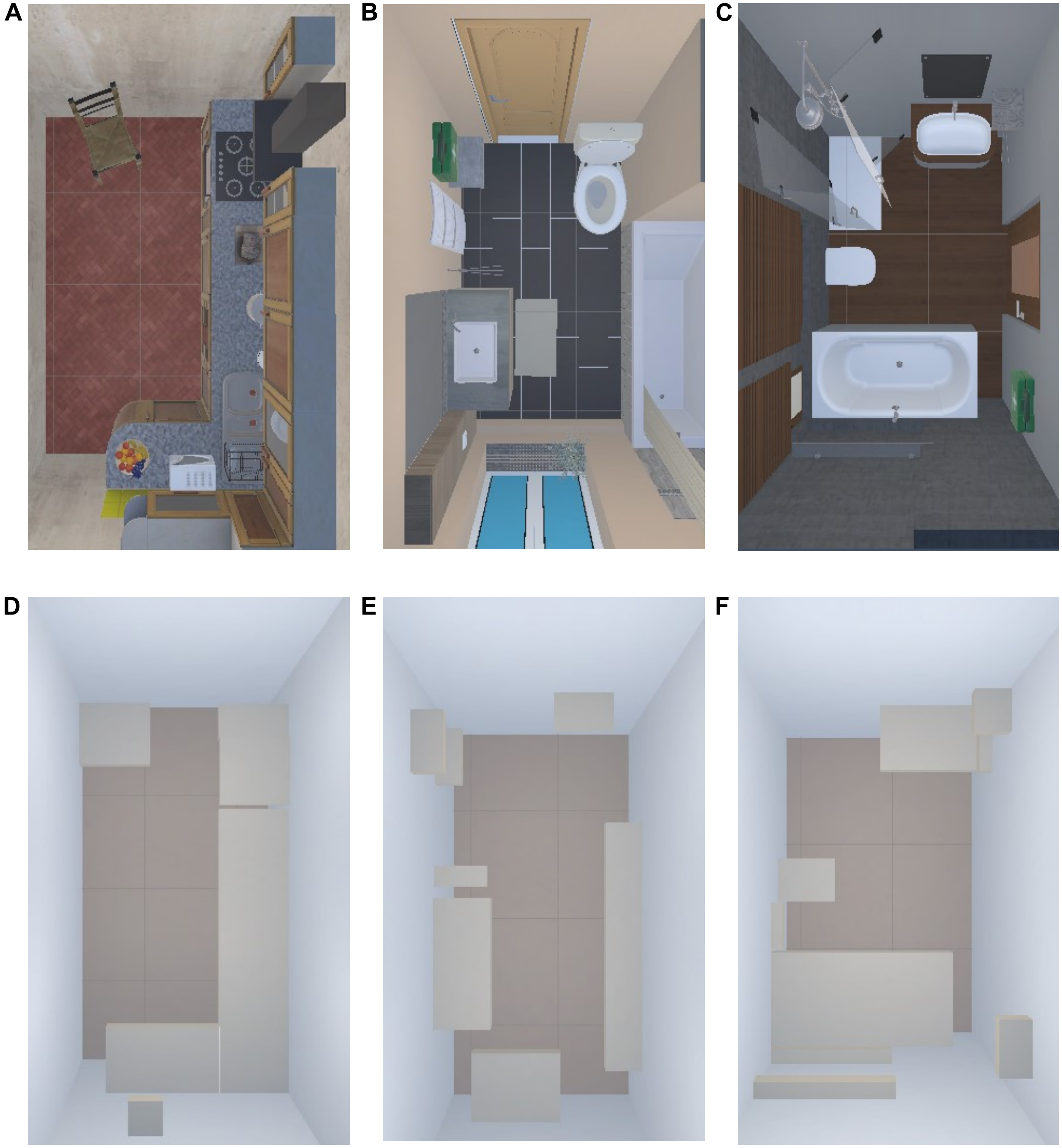

Figure S2: (**A**, **B**, **C**) the other three of the context-rich VR scenes from a total of six scenes, and (**D**, **E**, **F**) their empty matches. All scenes were designed using freely available 3D assets with CC0 or CC BY license (see Section S3).

Table S1: The target objects and their corresponding anchors used for the experiment.

Session	Object	Object type	Anchor	Context
training session	Street light	target object	-	-
training session	Park bench	target object	-	-
training session	Traffic light	target object	-	-
training session	Tree	target object	-	-
training session	Ladder	target object	-	-
training session	Scooter	target object	-	-
main experiment	Shampoo	target object	Bath tub	Bathroom
main experiment	Medical tape	target object	First aid kit	Bathroom
main experiment	Toilet paper	target object	Toilet	Bathroom
main experiment	Toothbrush	target object	Sink	Bathroom
main experiment	Toothpaste	target object	Sink	Bathroom
main experiment	Towel	target object	Towel holder	Bathroom
main experiment	Rubber duck	target object	Bath tub	Bathroom
main experiment	Dish washing sponge	target object	Sink	Kitchen
main experiment	Bread knife	target object	Bread on cutting board	Kitchen
main experiment	Microwave cover	target object	Microwave	Kitchen
main experiment	Pan	target object	Stove	Kitchen
main experiment	Plate	target object	Dish rack	Kitchen
main experiment	Pot lit	target object	Pot	Kitchen
main experiment	Tea cup	target object	Water boiler	Kitchen
main experiment	Paper	target object	Printer	Office
main experiment	Book	target object	Book shelf	Office
main experiment	Coffee jar	target object	Coffee machine	Office
main experiment	Computer mouse	target object	Computer / Laptop	Office
main experiment	Cushion	target object	Couch	Office
main experiment	Whiteboard duster	target object	Whiteboard	Office
main experiment	Whiteboard marker	target object	Whiteboard	Office

Table S2: Free 3D assets used to design the VR scenes for the experiment.

Object	Context	License	Source
Glass bottle	Kitchen	CC BY	source link
Kitchen	Kitchen	CC BY	source link
Kitchen	Kitchen	CC BY	source link
Dish washing sponge	Kitchen	CC BY	source link
Bread loaf	Kitchen	CC BY	source link
Bread loaf	Kitchen	CC BY	source link
Fruit basket	Kitchen	CC BY	source link
Microwave	Kitchen	CC BY	source link
Dish rack	Kitchen	CC BY	source link
Dish rack	Kitchen	CC BY	source link
Microwave	Kitchen	CC BY	source link
Bathroom	Bathroom	CC BY	source link
Shampoo bottles	Bathroom	CC BY	source link
Toilet	Bathroom	CC BY	source link
Outlet	Bathroom	CC BY	source link
Shelf	Bathroom	CC BY	source link
Bathroom	Bathroom	CC BY	source link
First aid box	Bathroom	CC BY	source link
First aid box	Bathroom	CC BY	source link
Desk	Office	CC BY	source link
Office chair	Office	CC BY	source link
Office chair	Office	CC BY	source link
Office lamp	Office	CC BY	source link
Computer	Office	CC BY	source link
Laptop	Office	CC BY	source link
Binder	Office	CC BY	source link
Shelf	Office	CC BY	source link
Printer	Office	CC BY	source link
Wall clock	Office	CC BY	source link
Notebook	Office	CC BY	source link
Small table	Office	CC BY	source link
Plants	Office	CC BY	source link
Couch	Office	CC BY	source link
Picture frame	Office	CC BY	source link
Paper bin	Office	CC BY	source link
Paper stand	Office	CC BY	source link
Notebook	Office	CC BY	source link
Armchair	Office	CC BY	source link
Printer	Office	CC BY	source link
Coffee machines	Office	CC BY	source link
Door	Office	CC BY	source link

Table S3: The results of the linear mixed model fitted to the task duration.
Formula in R: `LME <- lme(log(task duration) ~ condition, random = ~1|participant, data=data)`

Predictors	Estimate	SE	DF	t-value	p-value
(Intercept)	8.859431	0.03595373	1053	246.41202	0.000
congr-empty	0.008844	0.02352485	1053	0.37596	0.707
congr-incongruent	0.105849	0.02340395	1053	4.52268	0.000
Number of Observations: 1068					
AIC: 611.2019					
BIC: 636.0555					

Table S4: The results of the linear mixed model fitted to the search duration.
Formula in R: `LME <- lme(log(search duration) ~ condition, random = ~1|participant, data=data)`

Predictors	Estimate	SE	DF	t-value	p-value
(Intercept)	8.232600	0.03563829	1053	231.00434	0.0000
congr-empty	0.050603	0.04007937	1053	1.26257	0.2070
congr-incongruent	0.160530	0.03989124	1053	4.02420	0.0001
Number of Observations: 1068					
AIC: 1730.281					
BIC: 1755.135					

S4 Results of linear mixed model analysis

In Table S3 – Table S11 the results of the linear mixed models fitted to the data are shown. The models considered random intercept of the random factor. For data normalization, a logarithmic function was applied for all of the duration dependent variables, but not for the proportional variables.

Table S5: The results of the linear mixed model fitted to the search duration including the factor of the initial gaze-object distance.
Formula in R: `LME <- lme(log(search duration) ~ condition + initial gaze-object distance , random = ~1|participant, data=data)`

**Predictors**	**Estimate**	**SE**	**DF**	***t*-value**	***p*-value**
(Intercept)	7.801143	0.05929941	1052	131.55515	0.0000
congr-empty	0.035292	0.03843776	1052	0.91816	0.3587
congr-incongruent	0.158883	0.03821682	1052	4.15741	0.0000
initial gaze-object distance	0.361724	0.03762454	1052	9.61405	0.0000
Number of Observations: 1068					
AIC: 1649.099					
BIC: 1678.917					

Table S6: The results of the linear mixed model fitted to the reach duration.
Formula in R: `LME <- lme(log(reach duration) ~ condition, random = ~1|participant, data=data)`

**Predictors**	**Estimate**	**SE**	**DF**	***t*-value**	***p*-value**
(Intercept)	7.458410	0.05003607	1053	149.06067	0.0000
congr-empty	-0.025630	0.03873747	1053	-0.66164	0.5083
congr-incongruent	0.019025	0.03854101	1053	0.49364	0.6217
Number of Observations: 1068					
AIC: 1668.967					
BIC: 1693.821					

Table S7: The results of the linear mixed model fitted to the transport duration.
Formula in R: `LME <- lme(log(transport duration) ~ condition, random = ~1|participant, data=data)`

**Predictors**	**Estimate**	**SE**	**DF**	***t*-value**	***p*-value**
(Intercept)	7.379706	0.04935607	1053	149.51972	0.0000
congr-empty	-0.050494	0.01933753	1053	-2.61122	0.0092
congr-incongruent	-0.022732	0.01923634	1053	-1.18171	0.2376
Number of Observations: 1068					
AIC: 206.8258					
BIC: 231.6794					

Table S8: The results of the linear mixed model fitted to the scene coverage.
Formula in R: `LME <- lme(scene coverage ~ condition, random = ~1|participant, data=data)`

Predictors	Estimate	SE	DF	t-value	p-value
(Intercept)	0.022276896	0.0010247653	1053	21.738535	0.0000
congr-empty	0.000836529	0.0008834225	1053	0.946918	0.3439
congr-incongruent	0.004228697	0.0008789992	1053	4.810809	0.0000
Number of Observations: 1068					
AIC: -6387.093					
BIC: -6362.24					

Table S9: The results of the linear mixed model fitted to the proportion of gaze on target object.
Formula in R: `LME <- lme(proportion of gaze on target object ~ condition, random = ~1|participant, data=data)`

Predictors	Estimate	SE	DF	t-value	p-value
(Intercept)	0.30517600	0.013212741	1053	23.097099	0.0000
congr-empty	-0.00570256	0.007291331	1053	-0.782101	0.4343
congr-incongr	-0.01883927	0.007253536	1053	-2.597253	0.0095
Number of Observations: 1068					
AIC: -1879.358					
BIC: -1854.504					

Table S10: The results of the linear mixed model fitted to the proportion of gaze on anchor.
Formula in R: `LME <- lme(proportion of gaze on anchor ~ condition, random = ~1|participant, data=data)`

Predictors	Estimate	SE	DF	t-value	p-value
(Intercept)	0.09697857	0.004295039	707	22.579207	0.0000
congr-incongr	-0.01185809	0.004943283	707	-2.398829	0.0167
Number of Observations: 721					
AIC: -1833.385					
BIC: -1815.074					

Table S11: The results of the linear mixed model fitted to the anchor-object transition.
Formula in R: `LME <- lme(anchor-object transition ~ condition, random = ~1|participant, data=data)`

**Predictors**	**Estimate**	**SE**	**DF**	t**-value**	p**-value**
(Intercept)	31.04274	95.08595	679	0.3264703	0.7442
congr-incongr	282.96420	128.25806	679	2.2062098	0.0277
Number of Observations: 693					
AIC: 12252.59					
BIC: 12270.74					
